# Supplementary material for: Generalized leaky integrate-and-fire models classify multiple neuron types
Source: Nat Commun. 2018 Feb 19;9:709. doi: 10.1038/s41467-017-02717-4 (PMC5818568; doi:10.1038/s41467-017-02717-4)
Supplement: Supplementary file 1 — Supplementary Information [file 41467_2017_2717_MOESM1_ESM.pdf]

Supplementary Material for Generalized Leaky Integrate-And-Fire Models Classify Multiple Neuron Types

Corinne Teeter[1], Ramakrishnan Iyer[1], Vilas Menon[1,2], Nathan Gouwens[1], David Feng[1], Jim Berg[1], Aaron Szafer[1], Nicholas Cain[1], Hongkui Zeng[1], Michael Hawrylycz[1], Christof Koch[1], Stefan Mihalas[1]

December 20, 2017

1. Allen Institute for Brain Science 2. Howard Hughes Medical Institute, Janelia Research Campus

Correspondence and requests for materials should be addressed to CT (email: corinnet@alleninstitute.org) or SM (email: stefanm@alleninstitute.org)

Supplementary Figures

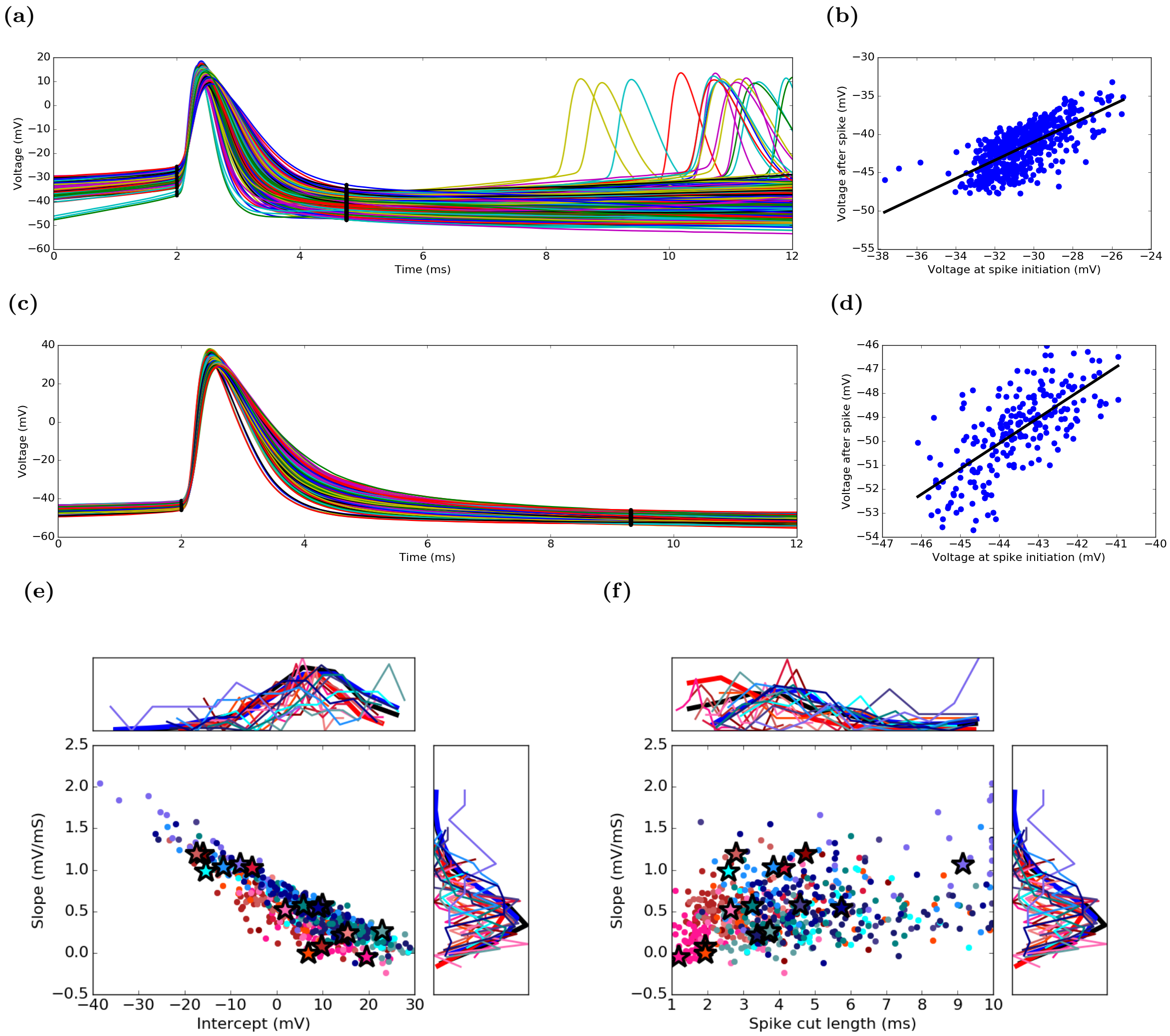

Figure 1: **The action potentials waveforms are excluded from model fitting but are used to derive voltage reset rules.** (a-d) illustrate examples of the spike cutting and reset rule calculation from two example neurons (a, b are from the Htr3a 474637203 neuron; c,d are from the Ctgf 512322162 neuron) from two different transgenic lines. (a, c) All spikes from the noise 1 stimuli (Figure 2 of the main article) are aligned to spike initiation. Here, different colors represent different spikes of one neuron (not different transgenic lines). Dots represent spike initiation and termination found by minimizing the residuals from a linear regression between pre- and post-spike voltage in a window 1 to 10 ms after spike initiation (b, d). In the ($GLIF_2$, $GLIF_4$, and $GLIF_5$ models, voltage reset is calculated by inserting the model voltage when it reaches threshold into the equation defined by the line (Equations 5 of the main text, and 4, 9, 12 in Supplementary Methods). (e) and (f) show distributions for slope, intercepts, and the spike cut length for all neurons. Normalized histograms of the data are shown in the side panels. All neurons with an intercept $<$ 30 mV were globally excluded from the data set. (f) is a replicate of the figure shown in the main article in Figure 3b.

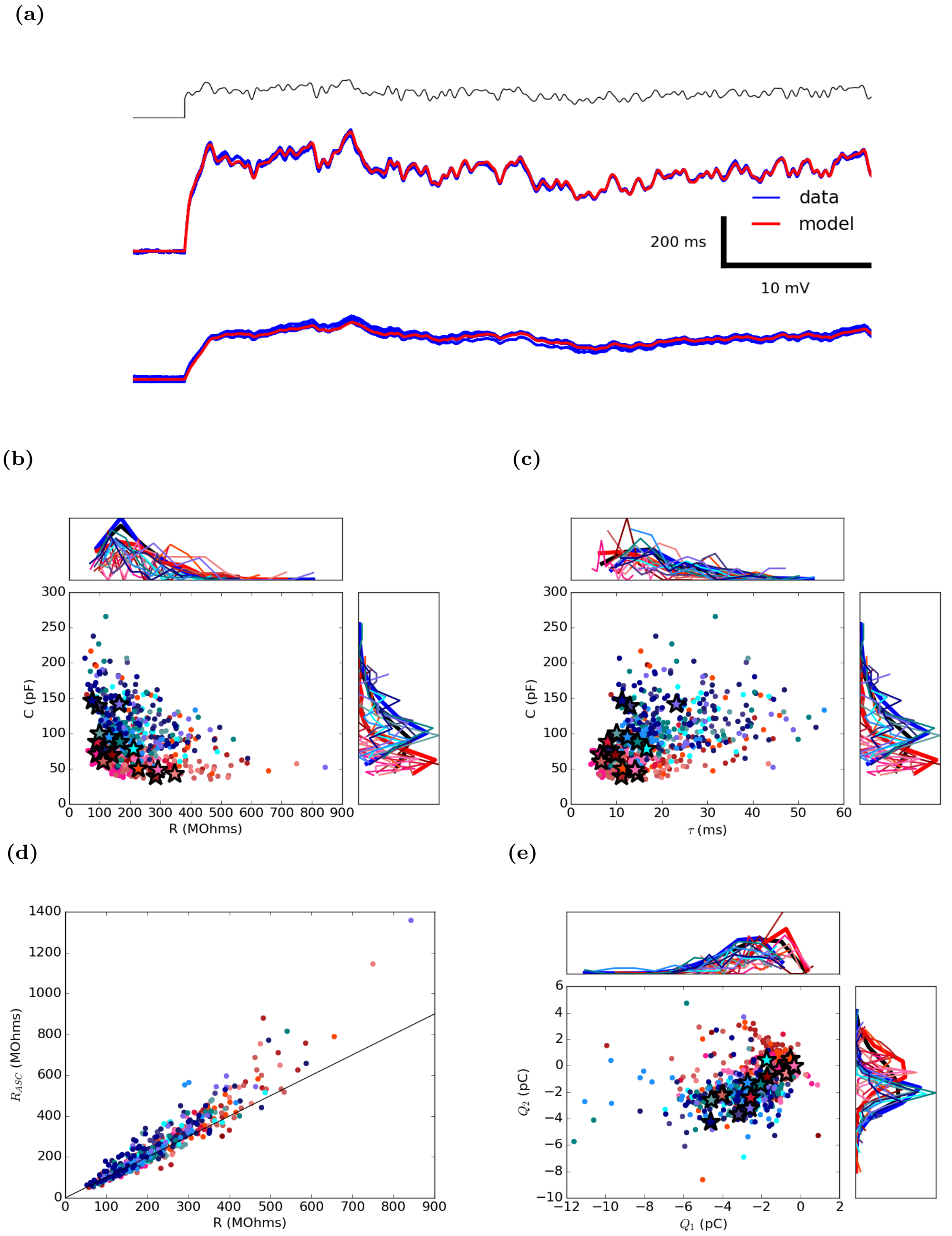

Figure 2: **Resistance, Capacitance and amplitudes of after-spike currents are fit via linear regression.** (a) Two examples of subthreshold voltage epochs fit via linear regression. Top traces are from the example cells shown in all figure examples (Top: Htr3a 474637203, Bottom: Ctgf 512322162). Injected current trace shown in black, voltage of biological neuron recorded from repeated current injections are in blue, voltage of model neuron red. Values of (b) resistance and capacitance and (c) the membrane time constant $\tau = RC$ and capacitance of all neurons fit to subthreshold data without the simultaneous fitting of after-spike currents. (d) Comparison of resistance values obtained by fitting subthreshold data without after-spike currents and supra-threshold data with the simultaneous fitting of after-spike currents. (e) Total charge deposited by after-spike currents. In scatter plots and corresponding distributions, shades of blue represent excitatory transgenic lines and red denote inhibitory transgenic lines. The thick black histograms represent data from all transgenic lines together, the thick red histograms represent data from inhibitory transgenic lines and the thick blue histogram represents data from excitatory transgenic lines. Stars denote example neurons as described in the main article. The full list of colors corresponding to specific transgenic lines can be found in Figure 2 of the main article. Any neuron containing a spike in the subthreshold noise epoch of the training set was globally eliminated from the data set. (c) and (e) are shown in main article in Figure 3c and 3d.

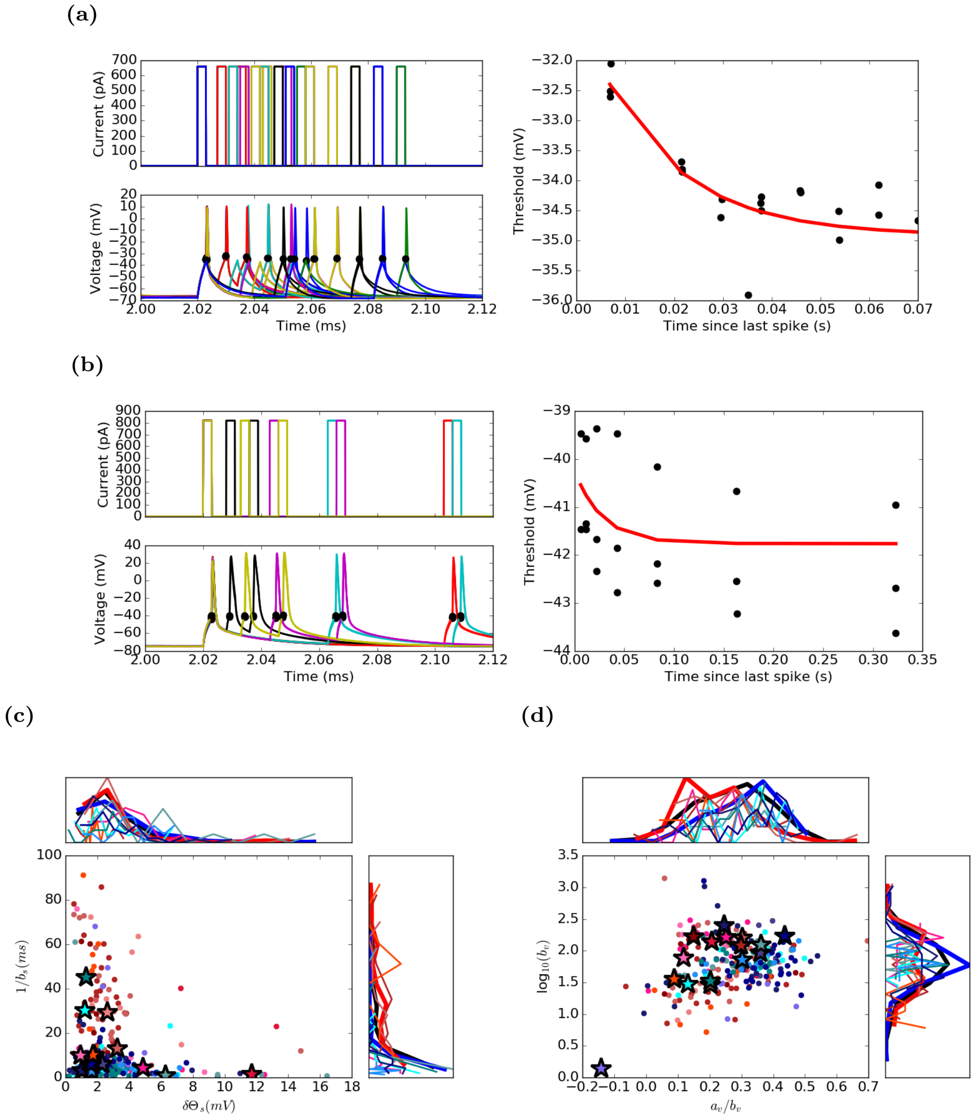

Figure 3: **The spiking component and voltage component of the threshold are incorporated into higher level models.** The spiking component of the threshold is defined by an exponential function fit to the time between spikes and the voltage at spike initiation of the triple short square data set. Exponential fitting of (a) Htr3a 474637203 and (b) Ctgf 512322162. Spiking in response to current pulses (left, with colors representing different sweeps) and exponential fit (right). Black dots denote spike initiation. (c) shows the fit amplitude, a, incorporated by the reset rule, δ_s, and the decay constant, b, of all neurons with triple square pulse data. All neurons with an $0 < a < 20mV$ or a $1/b > 0.1$ s are excluded. (d) In the $GLIF_5$ model the threshold is influenced by the voltage of the neuron. Parameters from equation 12 are plotted. $GLIF_5$ models where, $a_v < -50$, or, $b_v < 0.1$, are excluded. (c) and (d) are shown in main article, Figure 3e and 3f.

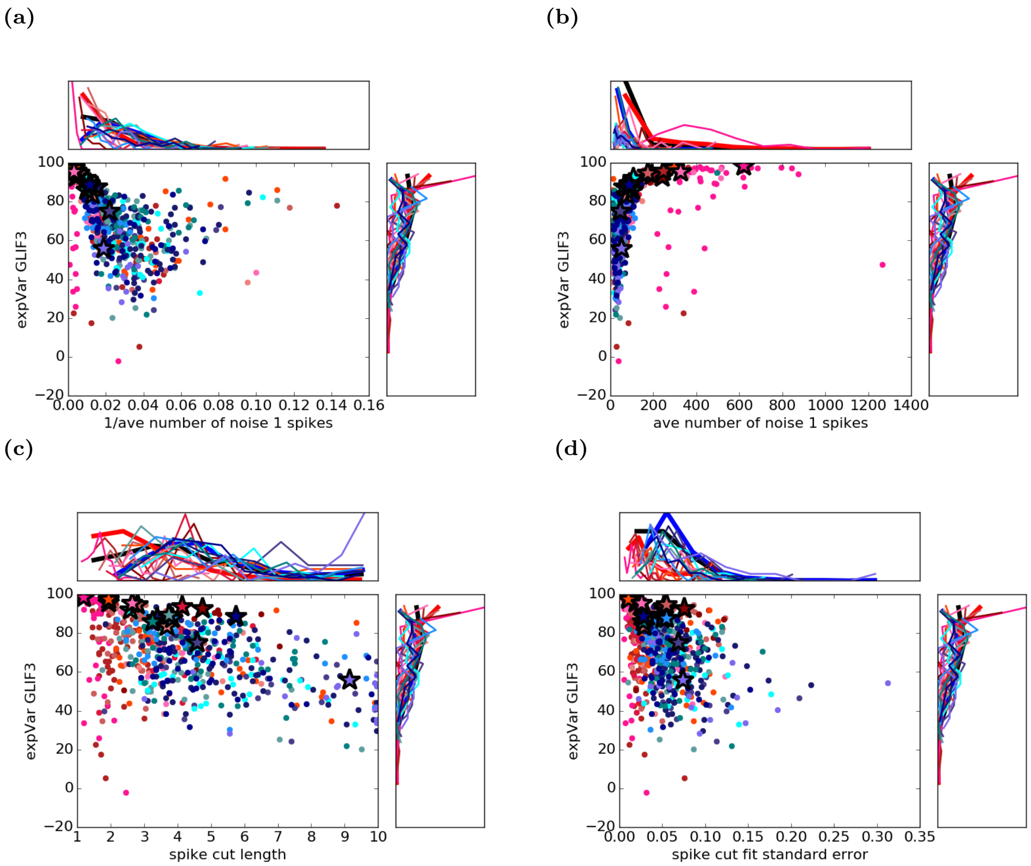

Figure 4: **Relationship between aspects of spiking and model performance.** In the main article, we report that models fit inhibitory neural data better than excitatory neural data. We then report that spike width and the number of spikes are important factors for predicting model performance whereas, spike reproducibility is not (as accessed by a multiple linear regression). Here we plot the data used in the multiple linear regression. (a) Plots the reciprocal of the average (average because the stimulus is repeated) number of spikes in each training (noise 1) data set versus the performance (assessed via the explained variance ratio). We used the reciprocal value because the non-reciprocal plot produces a quadratic-like output (b) and the multiple linear regression requires linear relationships to be interpretable. (c) Spike cut length (as defined in the "Parameter fitting and distributions" section of the Supplementary Methods and Supplementary Figure 1) versus the model performance of $GLIF_3$. (d) Spike cut length fit standard error versus the model performance of $GLIF_3$.

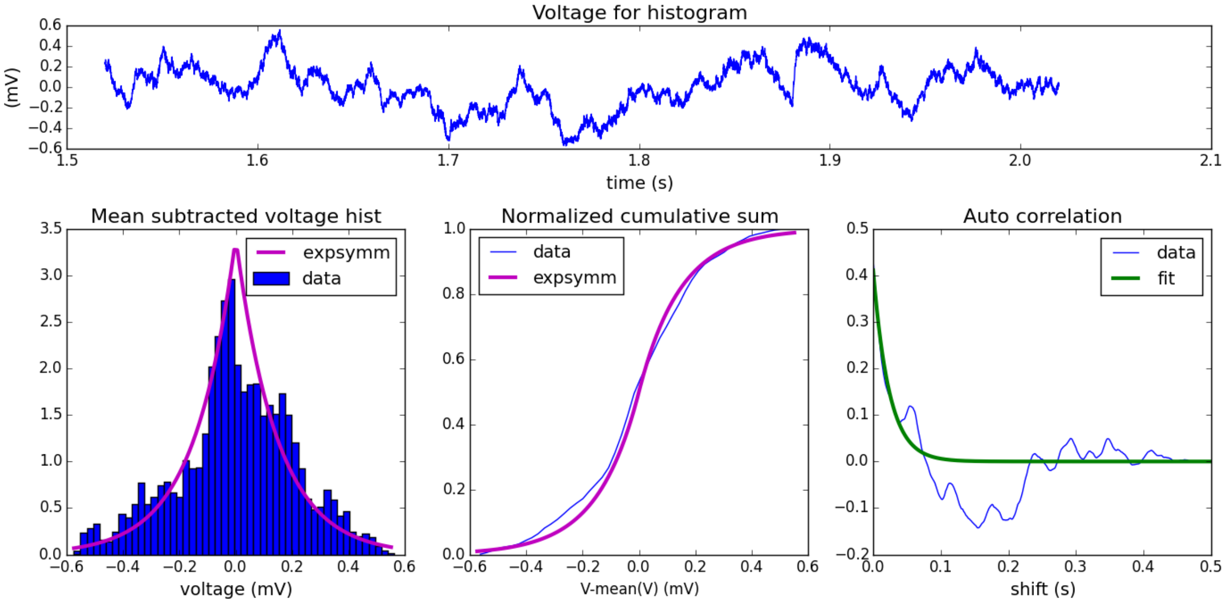

Figure 5: **Parameters of the MLIN objective function were extracted from the data.** A distribution of voltages is created from the tailing end of the largest amplitude subthreshold square pulse available (top panel). This distribution is then fit by a symmetrically decaying exponential (Supplementary Methods equation 34) function denoted as expsymm in the plot legends. The width of the non-spiking bins is chosen via a fit of the autocorrelation.

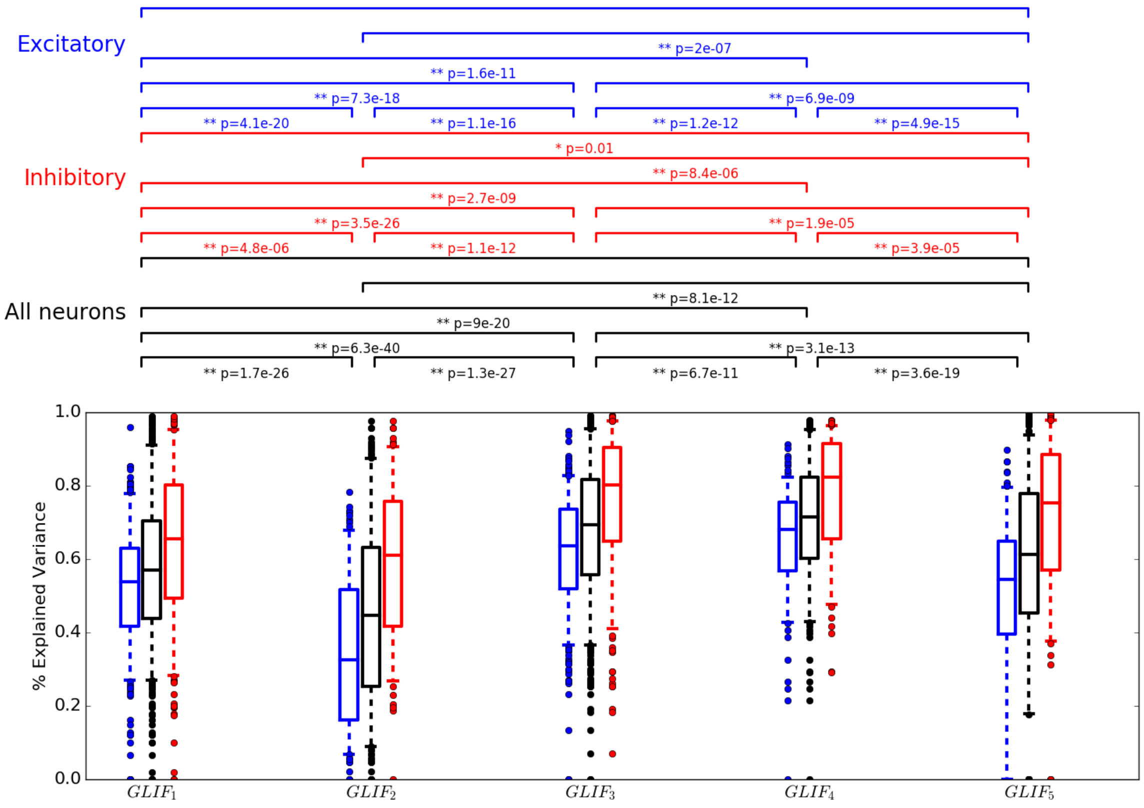

Figure 6: **Post-hoc Optimization of θ_∞ improves explained variance.** Explained variance summary for GLIF models for all 645 neurons (black), all 283 inhibitory neurons (red) and all 362 excitatory neurons (blue) for models with measured as opposed to post-hoc optimized θ_∞. Here average explained variance values are lower than after post-hoc optimization shown in Supplementary Figure 8. Box plots display median, and quartiles. Whiskers reach to 5% and 95% quantiles. Individual data points lie outside whiskers. Brackets above plot denote significant difference between distributions assessed via a Wilcoxon sign ranked test. P-values were corrected for the multiple comparison problem via the Benjamini-Hochberg procedure including all statistical tests with all transgenic lines (family size = 190). A single asterisk (*) represents a p-value < 0.05, a double asterisk (**) represents a p-value < 0.01.

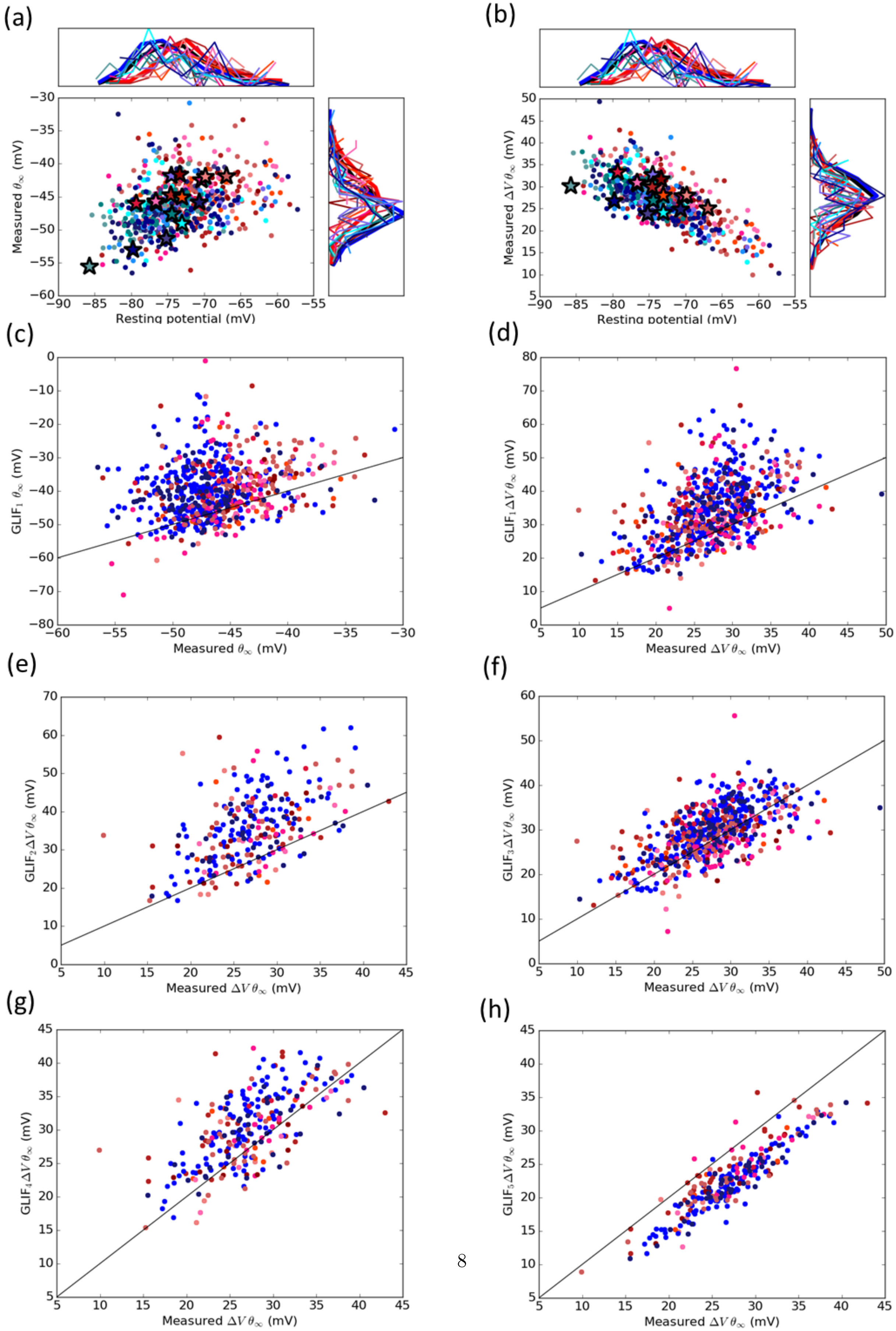
(a)
(b)
Measured θ_∞ (mV)
Resting potential (mV)
Measured $\Delta V\theta_\infty$ (mV)
Resting potential (mV)
(c)
(d)
GLIF$_1$ θ_∞ (mV)
Measured θ_∞ (mV)
GLIF$_1$ $\Delta V\theta_\infty$ (mV)
Measured $\Delta V\theta_\infty$ (mV)
(e)
(f)
GLIF$_2$ $\Delta V\theta_\infty$ (mV)
Measured $\Delta V\theta_\infty$ (mV)
GLIF$_3$ $\Delta V\theta_\infty$ (mV)
Measured $\Delta V\theta_\infty$ (mV)
(g)
(h)
GLIF$_4$ $\Delta V\theta_\infty$ (mV)
Measured $\Delta V\theta_\infty$ (mV)
GLIF$_5$ $\Delta V\theta_\infty$ (mV)
Measured $\Delta V\theta_\infty$ (mV)

Figure 7: **Resting potential and instantaneous threshold for biologically measured values and post-hoc optimized models**. In (a) absolute threshold is shown, in (b) threshold is in reference to the resting membrane potential for the biologically measured threshold from the short square stimulus (Figure 2 in the main article). This figure is also shown as Figure 3a in the main article. (c) and (d) show the similarity between threshold values before and after post-hoc optimization for absolute threshold (c) and relative threshold (d). (e), (f), (g), and (h) compare the biologically measured relative threshold versus the relative threshold obtained by optimizing the θ_∞ of (e) $GLIF_2$, (f) $GLIF_3$, (g), $GLIF_4$, and (h) $GLIF_5$. Black lines represents unity. Shades of blue represent excitatory, transgenic lines, shades of red represent inhibitory lines. The full list of colors corresponding to specific transgenic lines can be found in Figure 2 of the main article. Normalized histograms of the data are shown in the side panels. The thick black histogram represents data from all transgenic lines, the thick blue line represents all data from excitatory and the thick red represents inhibitory lines. Stars denote example neurons shown throughout the manuscript.

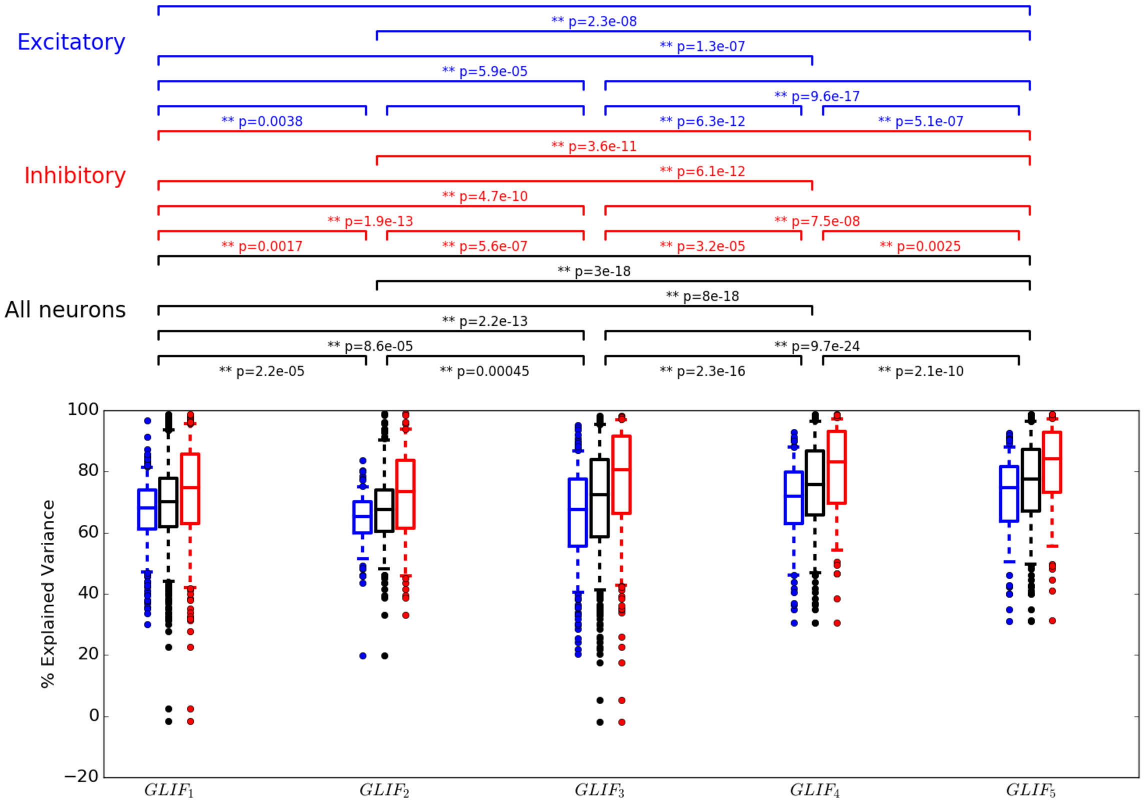

Figure 8: **Different mechanisms improve model performance for inhibitory and excitatory neurons.** Explained variance summary for GLIF models for all 645 neurons (black), all 283 inhibitory neurons (red) and all 362 excitatory neurons (blue). Box plots display medians, and quartiles. Whiskers reach to 5% and 95% quantiles. Individual data points lie outside whiskers. Brackets above plot denote significant difference between distributions assessed via a Wilcoxon sign ranked test. P-values were corrected for the multiple comparison problem via the Benjamini-Hochberg procedure including all statistical tests with all transgenic lines (family size = 190). A single asterisk (*) represents a p-value < 0.05, a double asterisk (**) represents a p-value < 0.01. Distribution values are available in Table 3 of the main article.

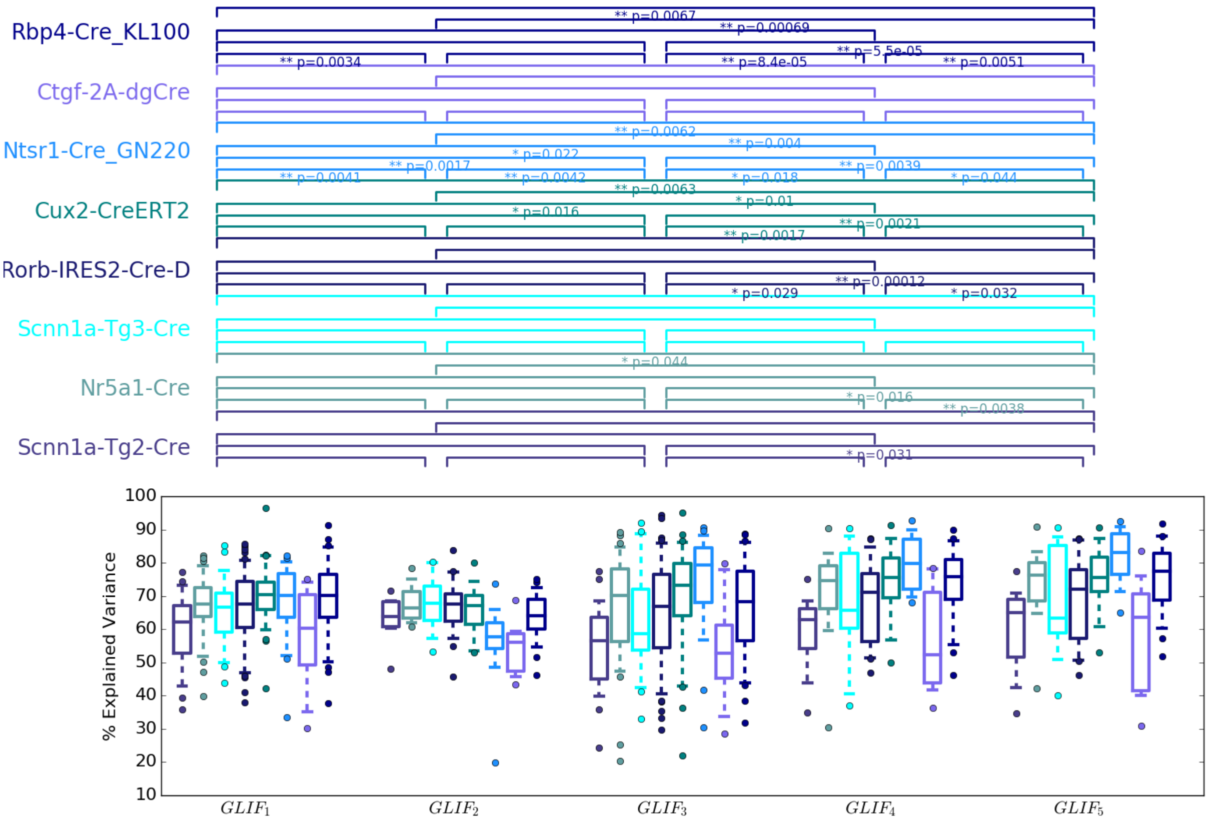

Figure 9: **Explained variance summary for excitatory GLIF models of different levels using threshold infinity obtained via MLIN optimization.** Box plots display medians, and quartiles. Whiskers reach to 5% and 95% quantiles. Individual data points lie outside whiskers. Brackets above plot denote significant difference between distributions assessed via a Wilcoxon sign ranked test. P-values were corrected for the multiple comparison problem via the Benjamini-Hochberg procedure including all statistical tests with all transgenic lines (family size = 190). A single asterisk (*) represents a p-value < 0.05, a double asterisk (**) represents a p-value < 0.01. Distribution values are available in Table 3 of the main article.

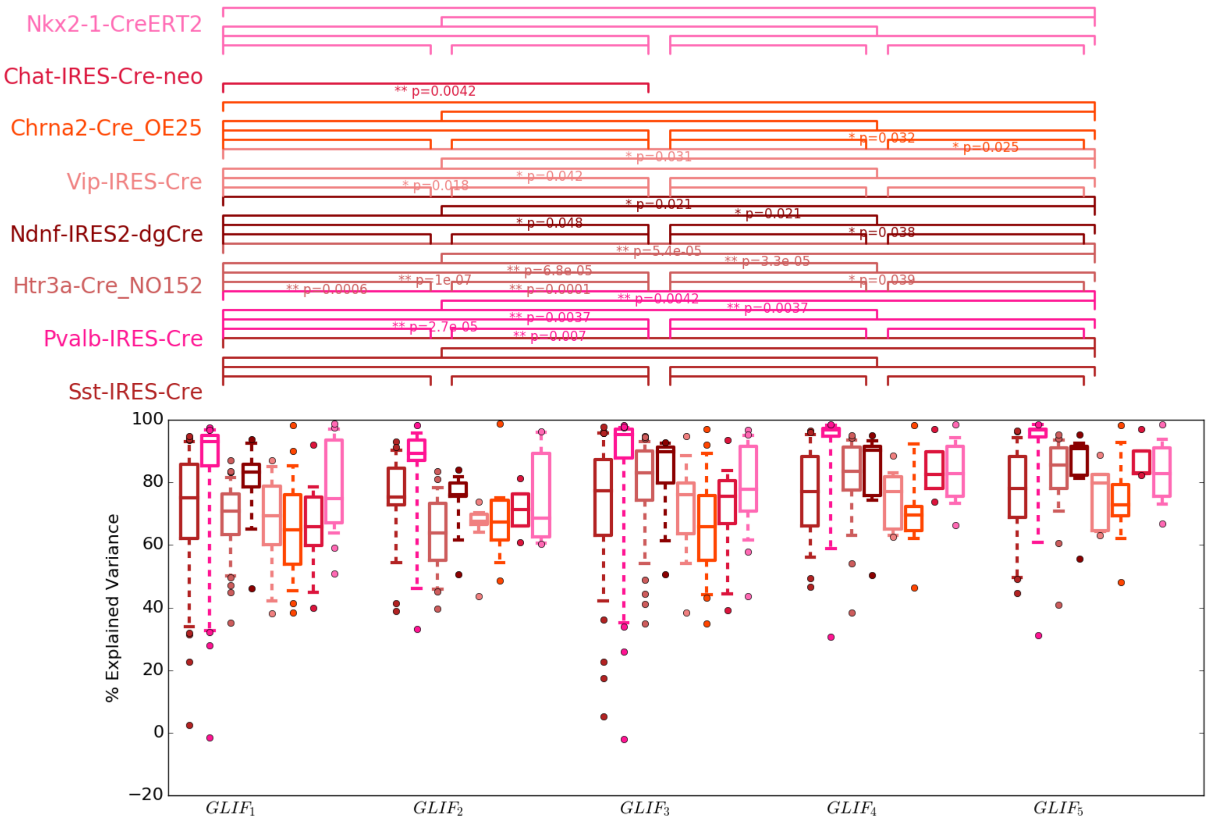

Figure 10: **Explained variance summary for inhibitory GLIF models of different levels using threshold infinity obtained via MLIN optimization.** Box plots display medians, and quartiles. Whiskers reach to 5% and 95% quantiles. Individual data points lie outside whiskers. Brackets above plot denote significant difference between distributions assessed via a Wilcoxon sign ranked test. P-values were corrected for the multiple comparison problem via the Benjamini-Hochberg procedure including all statistical tests with all transgenic lines (family size = 190). A single asterisk (*) represents a p-value < 0.05, a double asterisk (**) represents a p-value < 0.01. Distribution values are available in Table 3 of the main article.

(a)

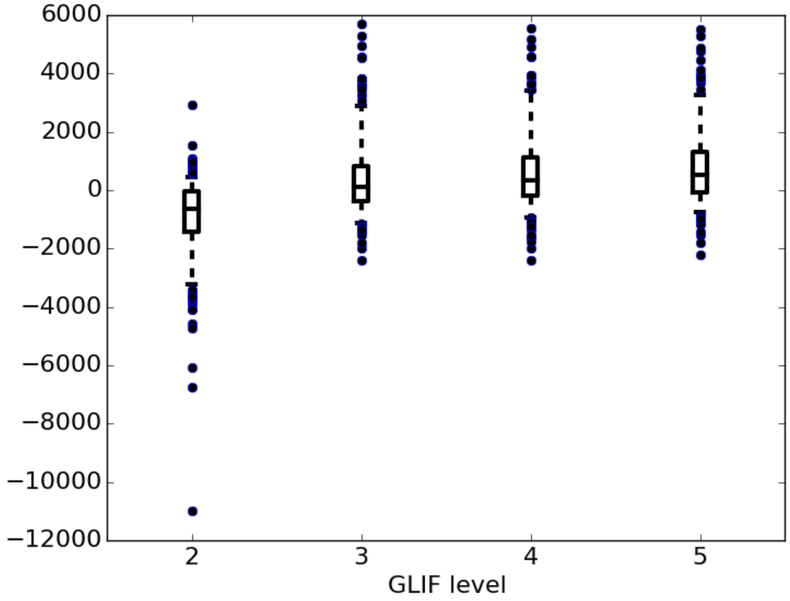

(b)

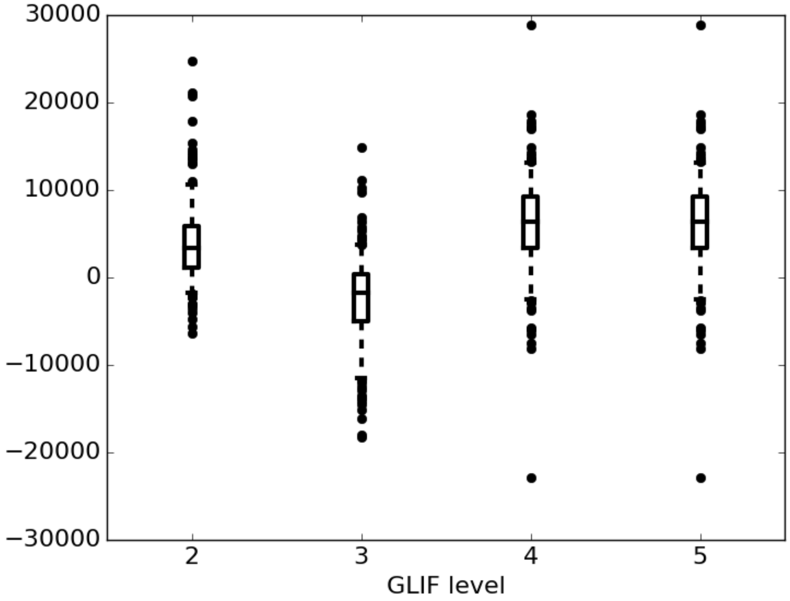

Figure 11: **Akaike Information Criterion (AIC) on 'training data' follows the same trend as explained variance ratio on 'hold out' data.** Difference between AIC for $GLIF_1$ and other $GLIF$ models is shown. Numbers on x-axis correspond to the $GLIF$ model compared to $GLIF_1$. a) Difference in AIC of model ability to reproduce spike times. b) Difference in AIC of model ability to reproduce subthreshold voltage behavior as defined in Supplementary Material Figure 15.

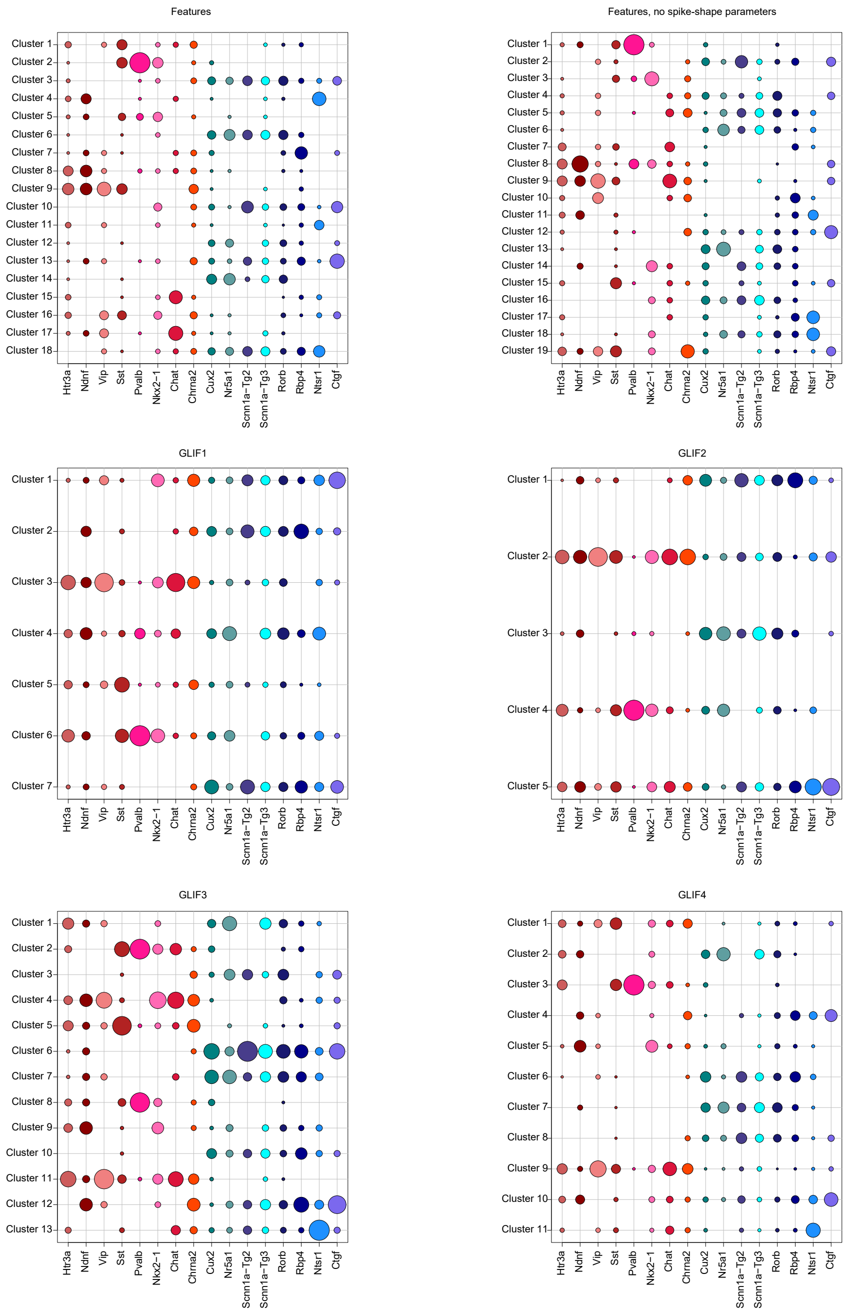
Features
Features, no spike-shape parameters
GLIF1
GLIF2
GLIF3
GLIF4
Htr3a
Ndnf
Vip
Sst
Pvalb
Nkx2−1
Chat
Chrna2
Cux2
Nr5a1
Scnn1a−Tg2
Scnn1a−Tg3
Rorb
Rbp4
Ntsr1
Ctgf

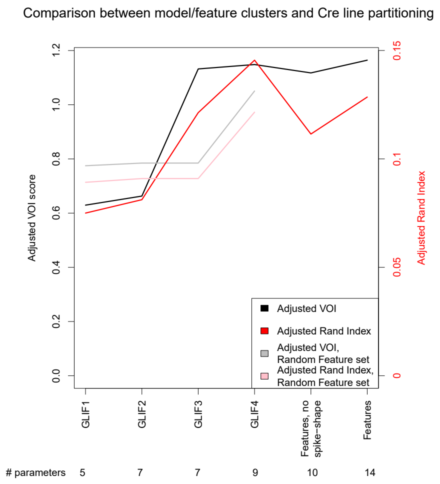
Comparison between model/feature clusters and Cre line partitioning
Adjusted VOI score
Adjusted Rand Index
Adjusted VOI
Adjusted Rand Index
Adjusted VOI, Random Feature set
Adjusted Rand Index, Random Feature set
GLIF1
GLIF2
GLIF3
GLIF4
Features, no spike−shape
Features
parameters
5
7
7
9
10
14

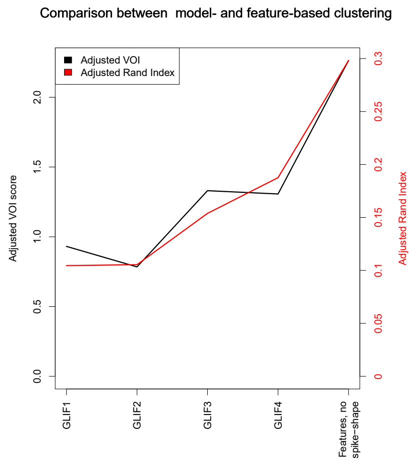
Comparison between model- and feature-based clustering
Adjusted VOI
Adjusted Rand Index
Adjusted VOI score
Adjusted Rand Index
GLIF1
GLIF2
GLIF3
GLIF4
Features, no spike−shape

Figure 12: **Clustering of cells using the affinity propagation method and the gap statistic to determine the optimal number of clusters.** The six panels show the segregation of clusters by transgenic line (labeled at the bottom of each panel) for each of the parameter sets used to cluster the cells. The bottom two panels show the Adjusted Variation of Information and Adjusted Rand Index scores for the clustering based on GLIF model and electrophysiological parameters, similar to Figure 6 in the main article.

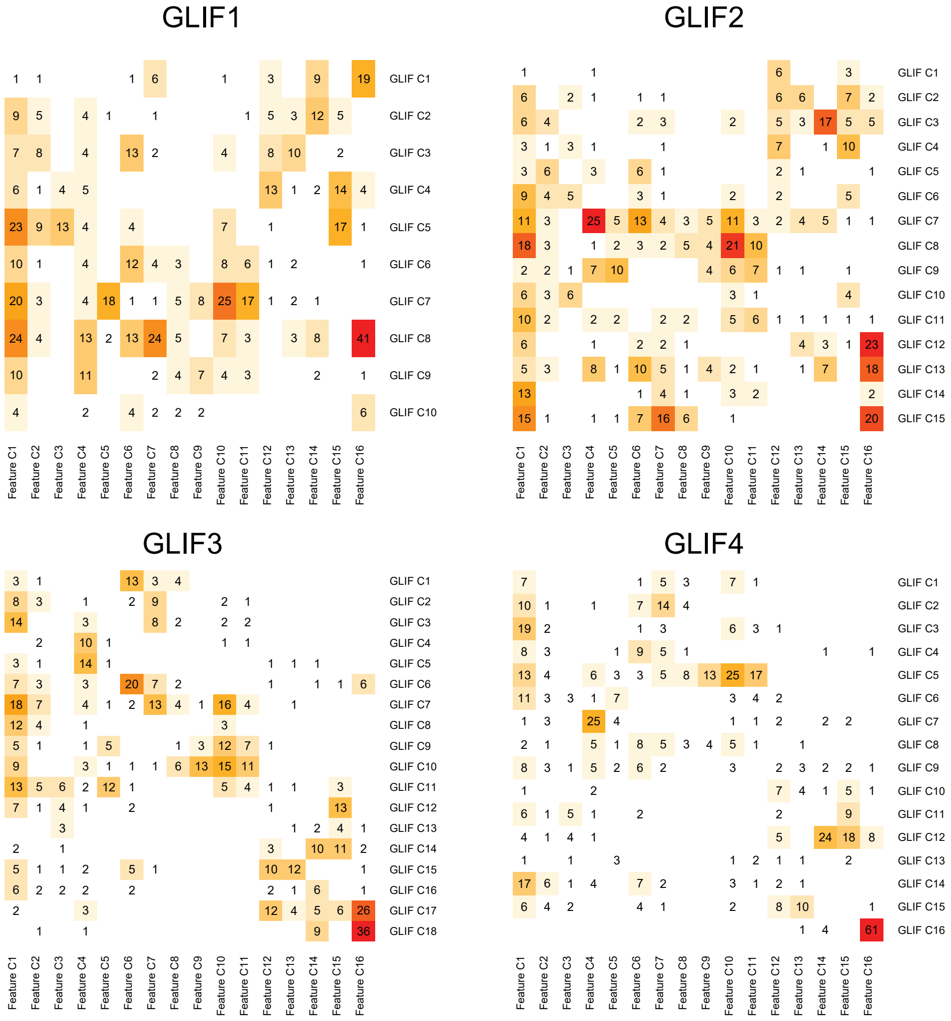

Figure 13: **Confusion matrices between the electrophysiological feature-based clustering (full set of 14 features) and the GLIF parameter-based clustering of cells using the iterative binary splitting method.** Each entry in each matrix represents the number of cells belonging to a specific feature cluster and GLIF cluster (Figure 6 of the main article), and indicated by the row and column labels. Zero values are represented as blank squares.

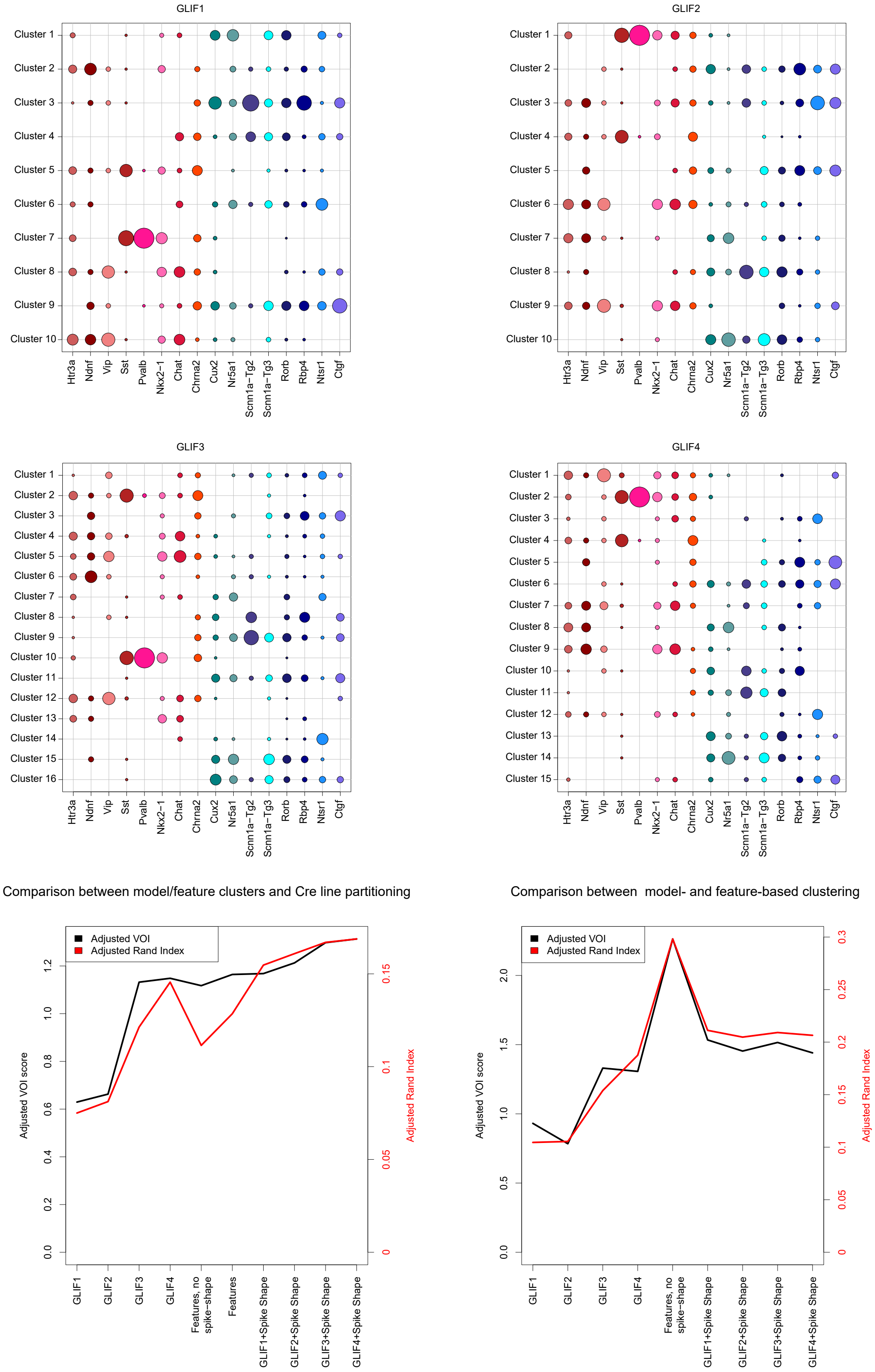

Figure 14: **Affinity propagation clustering obtained from using GLIF model parameters plus spike-shape-related feature parameters.** The top four panels show cluster versus Cre line composition, similar to Figure 7 of the main text. The bottom two panels show the Adjusted Variation of Information Metric and the Adjusted Rand Index for the GLIF model-derived clusters with and without the spike-shape parameters.

(a)

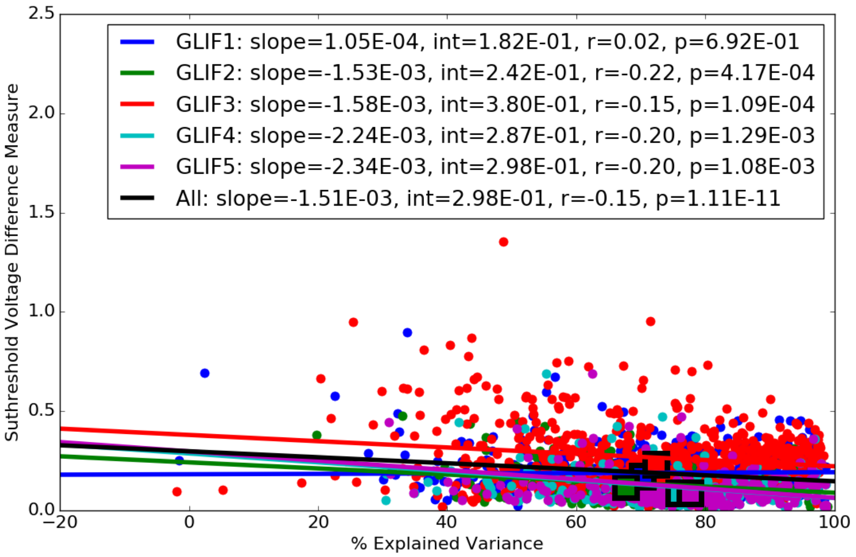

(b)

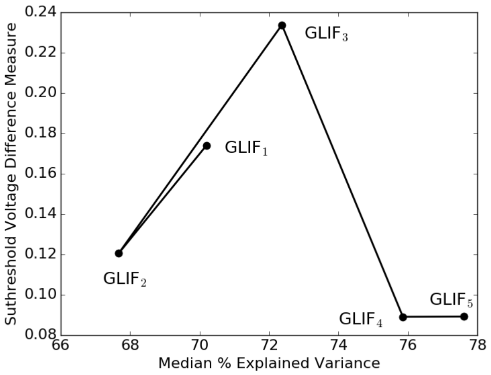

Figure 15: **Model levels which more accurately predict subthreshold voltage do not necessarily better predict spike times.** In a) overall, there is a correlation between the ability of models reproduce subthreshold voltage and reproduce spike times. Here, the percent explained variance (as shown in Table 3 and Figure 5 in the main article, and Supplementary Figure 8) is plotted against a measure of the difference in subthreshold voltage of the model and the neural voltage waveform. The "subthreshold voltage difference measure" is calculated by taking the residual sum of squares (RSS) between the data and neural model voltage normalized by the number of sampled time points considered, n. This is then normalized by the variance of the voltage of the neural data i.e. $(sum[(v_{data} - v_{model})^2]/n)/var(v_{data})$. Note that the smaller the value on the y-axes, the better the model is able to reproduce the subthreshold voltage. Because, the spike times of the model and the neural data are not perfectly aligned, the difference in subthreshold voltage was measured in the "forced-spike" paradigm as is done during the optimization procedure in Supplementary Methods section "Post-Hoc Optimization". Linear regression was performed on the GLIF levels separately and together. Slope, intercept, r, and p-values indicating the level of significance testing the hypothesis that the slope is not equal to zero are in the legend. Squares indicate median values for the GLIF levels. b) Median values pictured in a). The ability of the model to reproduce spike times does not translate into better fits of subthreshold voltage or the number of degrees of freedom of the model. Models with voltage reset rules fit directly from the voltage waveform (Supplementary Figure 1 better fit subthreshold behavior than $GLIF_1$ and $GLIF_3$ but more poorly recreate spiking behavior.

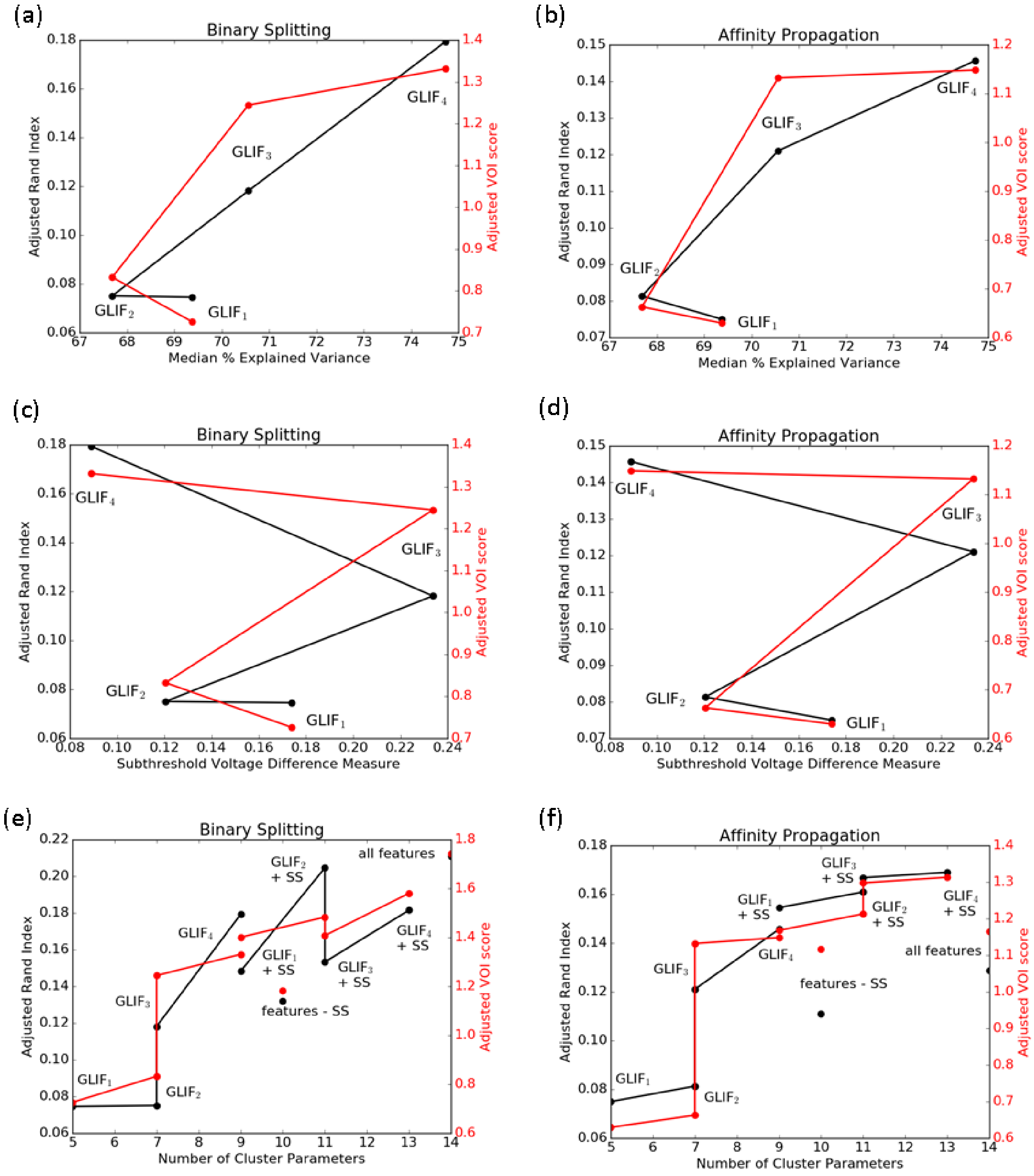

Figure 16: **Relationships between model performance and number of parameters and ability to differentiate Cre lines.** Y-axes on all plots show the ability of the parameters used to differentiate the different transgenic lines using the binary splitting and affinity propagation algorithms as measured by the adjusted rand index and adjusted variation of information as described in the text and all clustering figures. Solid lines accentuate the progression of GLIF model complexity. *SS*, abbreviates spike shape features. a) and b) show the median % explained variance metric as described in the text and is shown in Table 3 and Figure 5 of the main article, and Supplementary Figure 8. c) and d) show the medians "subthreshold voltage difference measure" described in Supplementary Figure 15. e) and f) show the number of parameters used during clustering.

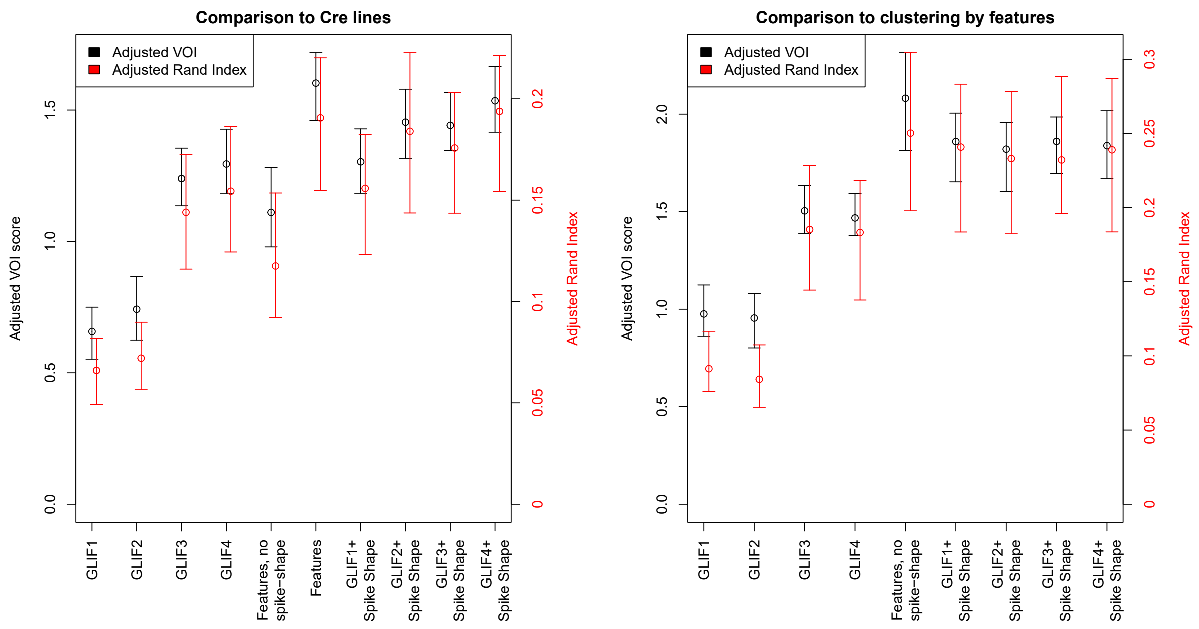

Figure 17: **Variability of clustering similarity using bootstrapping methods**. For each set of parameters (x-axes), 100 data subsets comprising a random selection of 80% of the cells were clustered using the iterative binary splitting method. For each bootstrapped data subset, the Adjusted VOI and Adjusted Rand Index were calculated, as described in the text. Circles represent the median values, and bars represent the 5th and 95th percentile values.

Supplementary Tables

Specimen ID	1	2	3	4	5
Pvalb					
515305346	0.935	0.984	0.926	0.984	0.986
481127173	0.945	0.895	0.971	0.971	0.968
477490421	0.976	0.958	0.983	**0.987**	0.985
484742372	0.929	0.871	0.960	0.965	0.963
Rorb					
320639930	0.708	0.674	0.656	0.718	0.732
473020156	0.639	0.708	0.702	0.718	0.727
502887600	0.645	0.597	0.556	0.559	0.577
500857324	0.590	0.627	0.582	0.631	0.755
313861677	0.672	0.688	0.432	0.562	0.544
485837504	0.620	0.657	0.548	0.706	0.680
467003163	0.706	0.622	**0.876**	0.873	0.873
480169178	0.657	0.775	0.772	0.767	0.806
478498617	0.559	0.652	0.673	0.663	0.682
502574847	0.747	0.717	0.770	0.776	0.778
480171386	0.695	0.723	0.575	0.516	0.597
501951290	0.739	0.662	0.480	0.597	0.605
480643417	0.767	0.772	0.759	0.747	0.767
480087928	0.658	0.677	0.490	0.469	0.462
Ntsr1					
485938494	0.796	0.524	0.898	0.880	0.925
488426904	0.755	0.572	0.882	0.901	0.902
485339983	0.726	0.650	0.692	0.755	0.801
485905411	0.612	0.545	0.772	0.776	0.778
490263438	0.816	0.622	0.876	**0.929**	0.910
322297406	0.631	0.543	0.646	0.682	0.649
Scnn1a-Tg3					
476557750	0.646	0.626	0.692	0.740	0.742
486893033	0.601	0.646	0.537	0.717	0.594
476263004	0.710	0.609	**0.889**	0.849	0.879
323838579	0.778	0.731	0.843	0.852	0.852
476269122	0.620	0.631	0.540	0.657	0.627
513832252	0.547	0.699	0.523	0.406	0.576
Ndnf					
579611597	0.834	0.756	0.875	0.884	0.885
527869035	0.865	0.840	0.912	0.916	0.916
530235049	0.811	**0.798**	0.917	0.908	0.907
Nr5a1					
502193385	0.733	0.714	0.551	0.689	0.708
318733871	0.638	0.665	0.582	0.735	0.739
502245101	0.741	0.785	0.593	0.772	**0.789**
489731121	0.691	0.655	0.601	0.764	0.766
321893798	0.503	0.627	0.458	0.304	0.423
Vip					
524876305	0.667	0.697	0.751	0.762	**0.778**
Cux2					
487358945	0.689	0.530	0.646	0.712	0.749
486239338	0.619	0.724	0.617	0.697	0.699
486198953	0.692	0.624	0.748	0.765	**0.765**
486146717	0.670	0.673	0.707	0.757	0.748
486108147	0.694	0.690	0.604	0.693	0.690
Htr3a					
488686369	0.688	0.653	0.877	0.884	0.886
475622793	0.633	0.565	**0.920**	0.919	0.918
519517188	0.749	0.682	0.878	0.833	0.849
482516216	0.727	0.742	0.841	0.833	0.851
487158599	0.750	0.653	0.893	0.826	0.856
475586130	0.685	0.663	0.806	0.776	0.796
473909767	0.625	0.589	0.762	0.736	0.781
Sst					
473593847	0.804	0.735	0.832	0.779	0.790
322197295	0.417	0.415	0.422	0.494	0.491
476686112	0.756	0.754	0.730	0.684	0.760
328015342	0.697	0.694	0.718	0.765	0.771
502382506	0.816	0.799	0.814	0.815	0.828
322229594	0.719	0.729	0.631	0.691	0.703
475057898	0.890	0.897	0.820	0.867	0.859
328031983	0.898	0.891	0.895	0.901	**0.903**
Rbp4					
485577686	0.685	0.641	0.711	0.759	0.757
471131311	0.766	0.707	0.714	0.758	**0.775**
508911693	0.563	0.648	0.549	0.644	0.667
490612844	0.683	0.597	0.683	0.709	0.719
485835016	0.637	0.548	0.645	0.686	0.710
485880739	0.664	0.584	0.544	0.700	0.629

Table 1: **Neurons which have all 5 GLIF models and all model parameters are within 5 and 95 percentiles within each transgenic line.** Columns 1 though 5 denote the explained variance of models 1 through 5. The best explained variance within the set is emboldened.

	R (MΩ)	τ (ms)	C (pF)	El (mV)	th_∞ (mV)	Δ th_∞ (mV)	$R_{ASC}(M\Omega)$	Q_1 (pC)
inhibitory	210<br>[143, 285]	13.2<br>[8.88, 19.3]	62.8<br>[52.4, 79.5]	-72.2<br>[-75.1, -68.9]	-45<br>[-47, -42.4]	26.9<br>[23.5, 30.2]	215<br>[147, 318]	-1.45<br>[-2.53, -0.808]
excitatory	177<br>[138, 237]	19<br>[14.9, 27.2]	107<br>[90.9, 131]	-75.5<br>[-78.2, -72.4]	-47.2<br>[-49, -45.4]	27.8<br>[24.9, 30.9]	190<br>[139, 277]	-2.91<br>[-3.97, -2.11]
Scnn1a-Tg2	201<br>[128, 232]	29.1<br>[19.5, 31.7]	135<br>[117, 159]	-74.9<br>[-76.6, -71.8]	-45.9<br>[-49.5, -44.3]	26.8<br>[25.7, 30.2]	249<br>[133, 321]	-3.5<br>[-4.5, -2.33]
Nr5a1	188<br>[153, 244]	17.9<br>[13.9, 24.8]	95.3<br>[87.1, 107]	-77.5<br>[-79.9, -74.6]	-47.3<br>[-48.8, -45.8]	30.8<br>[27.7, 32.1]	189<br>[133, 259]	-2.62<br>[-3.48, -2.24]
Scnn1a-Tg3	189<br>[151, 243]	21.9<br>[14.7, 27.9]	104<br>[89.8, 135]	-75.8<br>[-78, -72.9]	-46.4<br>[-48.1, -44.3]	29<br>[25.8, 32.8]	212<br>[147, 276]	-2.68<br>[-3.65, -1.63]
Rorb	194<br>[143, 265]	19.3<br>[15.7, 29.3]	101<br>[90, 125]	-75.6<br>[-78.5, -72.8]	-47.1<br>[-49.4, -45.1]	28.2<br>[25.2, 30.8]	208<br>[149, 300]	-2.89<br>[-3.8, -2.03]
Cux2	163<br>[135, 207]	16.8<br>[14, 21.7]	108<br>[91.4, 122]	-75.6<br>[-78.3, -73.4]	-47.5<br>[-48.4, -45.9]	28.2<br>[26.3, 30.8]	167<br>[137, 210]	-2.92<br>[-4.09, -2.19]
Ntsr1	174<br>[150, 219]	16.2<br>[14, 19.1]	94.1<br>[85.6, 106]	-75.5<br>[-77.9, -71.9]	-47.5<br>[-48.5, -45.3]	27.6<br>[25.5, 30.4]	173<br>[142, 231]	-3.03<br>[-5.27, -2.35]
Ctgf	247<br>[164, 351]	26.3<br>[22.5, 37.2]	118<br>[96.5, 136]	-71.8<br>[-75.5, -67.5]	-46<br>[-47.9, -44.6]	24.8<br>[19.1, 28.4]	304<br>[195, 439]	-2.46<br>[-3.16, -1.88]
Rbp4	138<br>[116, 186]	18.2<br>[14.1, 24.2]	131<br>[111, 150]	-74.2<br>[-77.7, -68.9]	-47.9<br>[-49.3, -46]	25<br>[22.5, 28.8]	162<br>[124, 241]	-3.11<br>[-4.53, -2.19]
Sst	226<br>[168, 311]	16.5<br>[11.2, 23.5]	71<br>[62, 88.7]	-71.5<br>[-73.9, -69.2]	-46<br>[-48.5, -43.5]	24.5<br>[22.4, 28.4]	236<br>[174, 335]	-1.37<br>[-1.79, -1.01]
Pvalb	126<br>[103, 176]	7.13<br>[6.5, 8.84]	54.4<br>[49.4, 65.6]	-75.2<br>[-76.8, -72.3]	-46.2<br>[-47.9, -43.3]	28<br>[26.4, 30.8]	131<br>[108, 177]	-0.734<br>[-1.2, -0.515]
Htr3a	206<br>[134, 335]	11.6<br>[7.91, 17]	55.2<br>[44.9, 66.5]	-71.6<br>[-73.8, -67.4]	-43.9<br>[-46, -40.6]	26.6<br>[23.9, 30.7]	212<br>[144, 352]	-2.24<br>[-3.98, -1.02]
Ndnf	183<br>[137, 212]	12.4<br>[11.3, 14.2]	69.3<br>[60.2, 87]	-72.5<br>[-74.4, -67.9]	-42.7<br>[-44.3, -41.2]	28.7<br>[24.2, 32.3]	197<br>[147, 233]	-2.04<br>[-2.55, -1.76]
Chat	224<br>[211, 307]	14.1<br>[12.2, 16]	55.9<br>[54, 66.2]	-73.4<br>[-75, -70.3]	-46.9<br>[-47.7, -45.3]	25.9<br>[23.6, 28.6]	245<br>[218, 314]	-2.34<br>[-2.71, -1.85]
Vip	332<br>[252, 411]	19.9<br>[15.7, 21.5]	57.9<br>[48.6, 72.5]	-72<br>[-73.5, -67]	-46<br>[-47.2, -43.6]	25.4<br>[22.9, 27.6]	374<br>[246, 516]	-1.28<br>[-2.49, -0.772]
Chrna2	279<br>[195, 362]	25.9<br>[18, 31.9]	90.2<br>[71, 126]	-70.8<br>[-73.3, -66.4]	-44.3<br>[-46, -42.5]	25.8<br>[21.1, 29.6]	295<br>[206, 415]	-2.35<br>[-3.4, -1.09]
Nkx2	223<br>[208, 249]	13.4<br>[12.3, 16]	64.5<br>[60.4, 70.9]	-69.6<br>[-73.3, -66.4]	-41.3<br>[-43.9, -38.7]	28.8<br>[25, 31.8]	243<br>[230, 277]	-2.28<br>[-3.61, -0.496]

Table 2: **Summary characterization of GLIF parameters for different transgenic lines**. The single number in each cell is the median. The low and high quartiles are specified in brackets below the median. Some neurons have the required stimuli for LIF models but do not have the stimuli required for higher level models. When there are not more than five neurons that have the parameter, these values are denoted with a NaN.

	Q_2 (pC)	δt (ms)	slope	intercept (mV)	a_{spike} (V)	$1/(b_{spike})$ (s)	$(a_v)/(b_v)$	$log_{10}(b_v)$
inhibitory	-0.278<br>[-0.925, 0.682]	2.65<br>[1.9, 3.73]	0.419<br>[0.223, 0.607]	5.87<br>[-0.766, 10.8]	0.00232<br>[0.00172, 0.00322]	0.0219<br>[0.00826, 0.0396]	0.226<br>[0.141, 0.295]	1.75<br>[1.43, 2.17]
excitatory	-2.12<br>[-2.71, -1.6]	4.6<br>[3.65, 6.04]	0.483<br>[0.27, 0.727]	8.6<br>[1.03, 15.9]	0.00266<br>[0.00162, 0.00389]	0.00414<br>[0.00192, 0.00757]	0.329<br>[0.234, 0.387]	1.83<br>[1.64, 2.02]
Scnn1a-Tg2	-2.32<br>[-3.64, -1.87]	6.55<br>[4.8, 7.65]	0.536<br>[0.345, 0.623]	8.86<br>[3.03, 14.1]	0.00103<br>[0.000518, 0.00171]	0.00225<br>[0.00104, 0.00374]	0.359<br>[0.353, 0.4]	2<br>[1.88, 2.17]
Nr5a1	-1.97<br>[-2.37, -1.71]	3.7<br>[2.85, 4.53]	0.248<br>[0.0962, 0.391]	20.5<br>[11.2, 24.3]	0.00488<br>[0.00296, 0.00679]	0.00153<br>[0.00121, 0.0038]	0.294<br>[0.255, 0.358]	1.89<br>[1.77, 2.01]
Scnn1a-Tg3	-2.07<br>[-2.48, -1.76]	4.85<br>[3.95, 6.2]	0.375<br>[0.192, 0.597]	12.4<br>[3.09, 19.5]	0.00222<br>[0.00169, 0.00426]	0.00353<br>[0.0018, 0.00849]	0.356<br>[0.197, 0.42]	1.88<br>[1.64, 2.05]
Rorb	-1.99<br>[-2.37, -1.59]	4.75<br>[3.74, 6.21]	0.392<br>[0.249, 0.553]	10.3<br>[4.91, 16.8]	0.002<br>[0.00129, 0.00321]	0.0037<br>[0.00228, 0.00767]	0.285<br>[0.195, 0.352]	1.85<br>[1.61, 2.02]
Cux2	-2.03<br>[-2.6, -1.69]	4.62<br>[3.6, 5.81]	0.422<br>[0.261, 0.586]	11.8<br>[7.43, 15.8]	0.00286<br>[0.002, 0.00321]	0.00402<br>[0.00147, 0.00588]	0.29<br>[0.231, 0.353]	1.81<br>[1.76, 1.91]
Ntsr1	-2.21<br>[-2.96, -1.27]	3.9<br>[3.3, 5.55]	0.764<br>[0.624, 0.948]	-1.67<br>[-6.84, 5.84]	0.00434<br>[0.00345, 0.00571]	0.00299<br>[0.00274, 0.00471]	0.313<br>[0.23, 0.379]	1.81<br>[1.73, 1.93]
Ctgf	-1.96<br>[-2.72, -0.859]	8.78<br>[6.26, 9.91]	1.21<br>[0.94, 1.45]	-11.3<br>[-23.1, -3.61]	0.00231<br>[0.00192, 0.00326]	0.0114<br>[0.00855, 0.0454]	0.24<br>[0.226, 0.318]	1.41<br>[1.03, 1.48]
Rbp4	-2.49<br>[-3.08, -1.19]	4.55<br>[3.75, 5.4]	0.683<br>[0.474, 0.848]	4.01<br>[-2.16, 10.8]	0.00263<br>[0.00181, 0.00403]	0.00673<br>[0.00411, 0.00883]	0.378<br>[0.325, 0.428]	1.74<br>[1.6, 1.95]
Sst	0.958<br>[0.484, 1.57]	2.1<br>[1.85, 2.62]	0.488<br>[0.326, 0.62]	0.956<br>[-4.14, 4.74]	0.00206<br>[0.00147, 0.00277]	0.0345<br>[0.0232, 0.0439]	0.247<br>[0.162, 0.312]	1.49<br>[1.34, 1.92]
Pvalb	0.0909<br>[-0.395, 0.553]	1.65<br>[1.44, 1.8]	0.162<br>[0.0439, 0.273]	11.1<br>[7.56, 15.2]	0.00365<br>[0.0026, 0.00486]	0.00374<br>[0.00262, 0.00623]	0.195<br>[0.15, 0.257]	2.19<br>[2.04, 2.29]
Htr3a	-0.57<br>[-1.6, 0.227]	2.75<br>[2.38, 3.05]	0.456<br>[0.357, 0.606]	6.36<br>[-0.766, 11.3]	0.00245<br>[0.00207, 0.00303]	0.0212<br>[0.0122, 0.0365]	0.228<br>[0.153, 0.299]	1.81<br>[1.5, 2.2]
Ndnf	-1.38<br>[-1.95, -0.897]	4.7<br>[4.25, 5.15]	0.351<br>[0.226, 0.774]	9.54<br>[-4.34, 14.8]	0.00182<br>[0.00174, 0.00264]	0.0113<br>[0.00979, 0.0287]	0.134<br>[0.12, 0.254]	1.81<br>[1.6, 2.11]
Chat	-0.376<br>[-1.05, 0.448]	4.15<br>[3.93, 4.49]	0.637<br>[0.468, 0.682]	4.52<br>[2.73, 9.16]	NaN	NaN	NaN	NaN
Vip	-0.485<br>[-0.642, -0.176]	3.75<br>[3.53, 4.5]	0.47<br>[0.35, 0.734]	4.15<br>[1.1, 8.8]	0.00302<br>[0.00232, 0.00424]	0.0342<br>[0.031, 0.0584]	0.22<br>[0.148, 0.303]	1.51<br>[1.29, 1.83]
Chrna2	-0.377<br>[-2.09, 0.889]	4.65<br>[2.9, 6.65]	0.428<br>[0.282, 0.68]	4.79<br>[-3.2, 7.35]	0.00175<br>[0.00166, 0.00199]	0.035<br>[0.0112, 0.0541]	0.243<br>[0.127, 0.309]	1.47<br>[1.12, 1.67]
Nkx2	-0.553<br>[-1.34, -0.387]	3.55<br>[2.65, 3.88]	0.373<br>[0.125, 0.589]	6.44<br>[-0.41, 11.9]	NaN	NaN	NaN	NaN

Table 3: **SECOND HALF OF TABLE 2**

	R (MΩ)	τ (ms)	C (pF)	El (mV)	th_∞ (mV)	Δ th_∞ (mV)	$R_{ASC}(M\Omega)$	Q_1 (pC)
1	141<br>[121, 153]	15.5<br>[13.8, 16.6]	111<br>[104, 117]	-77<br>[-78.8, -75.5]	-48.3<br>[-50.2, -46]	29.1<br>[26.7, 30]	129<br>[110, 147]	-3.49<br>[-3.91, -2.77]
2	159<br>[119, 189]	13.9<br>[10.4, 17.1]	90.3<br>[81.9, 94.9]	-79.5<br>[-81.4, -77.6]	-47.4<br>[-49.4, -44.3]	32<br>[30.8, 34.9]	149<br>[117, 185]	-2.81<br>[-4.12, -2.23]
3	172<br>[143, 203]	15.6<br>[12.6, 20.4]	91.7<br>[84.1, 103]	-74.6<br>[-77.8, -72.9]	-47.5<br>[-48.7, -45.9]	27.4<br>[25.1, 28.8]	175<br>[140, 211]	-2.04<br>[-2.35, -1.44]
4	196<br>[153, 237]	14.3<br>[8.69, 19.2]	66.4<br>[60.7, 87.4]	-77.2<br>[-80.8, -73.5]	-43.9<br>[-46.2, -41.6]	31.1<br>[29.8, 36]	196<br>[153, 261]	-2.58<br>[-3.77, -2.2]
5	159<br>[115, 207]	20.4<br>[15.6, 28.9]	137<br>[116, 159]	-76.4<br>[-78.2, -74.3]	-47.8<br>[-49.2, -46.5]	28.7<br>[25, 30.7]	158<br>[113, 239]	-4.05<br>[-5.02, -3.23]
6	170<br>[123, 263]	23.8<br>[17.9, 30.3]	127<br>[112, 151]	-68.2<br>[-70.8, -63.7]	-47.3<br>[-49.5, -44.8]	21.3<br>[17.7, 23.2]	212<br>[163, 320]	-3<br>[-3.4, -2.05]
7	173<br>[153, 203]	17.6<br>[14.7, 19.4]	101<br>[88.5, 114]	-74.5<br>[-77.5, -71.7]	-47.2<br>[-48.7, -45.1]	25.9<br>[24.5, 29.5]	190<br>[147, 211]	-3.37<br>[-6.07, -2.52]
8	139<br>[116, 197]	14.6<br>[11.1, 19.5]	93<br>[82.1, 113]	-77.4<br>[-78.8, -75.2]	-45<br>[-48.1, -43.2]	31.6<br>[27.8, 36]	150<br>[118, 233]	-3.24<br>[-4.15, -2.75]
9	239<br>[182, 292]	16.1<br>[13.1, 26]	80.7<br>[60.4, 98.6]	-69.6<br>[-73.4, -65.7]	-43<br>[-46.6, -39.5]	26.7<br>[21.6, 30.4]	249<br>[196, 353]	-1.88<br>[-2.28, -1.51]
10	361<br>[334, 462]	15.9<br>[14.6, 19.6]	44.6<br>[42.1, 48.5]	-72.1<br>[-73.5, -69.9]	-45<br>[-47.7, -42.3]	26.2<br>[24.5, 30.4]	408<br>[343, 584]	-0.725<br>[-1.04, -0.51]
11	406<br>[273, 518]	21.7<br>[18.8, 33.4]	61.2<br>[50.3, 75.8]	-65.5<br>[-68.3, -63.3]	-44.3<br>[-46.6, -41.6]	21.9<br>[20.4, 24.5]	525<br>[309, 564]	-1.1<br>[-1.59, -0.722]
12	225<br>[184, 292]	18.6<br>[13.7, 25.1]	79.2<br>[66.6, 91.3]	-71.4<br>[-73.3, -67.8]	-45.6<br>[-47.1, -43.4]	24.7<br>[22.1, 27.2]	243<br>[189, 318]	-1.49<br>[-1.95, -1.1]
13	185<br>[149, 264]	21.2<br>[17.2, 27.7]	120<br>[91.9, 144]	-69.2<br>[-71.2, -66.7]	-47.2<br>[-48.4, -46.1]	22.6<br>[18.8, 24.4]	215<br>[175, 319]	-3.72<br>[-4.71, -2.68]
14	268<br>[215, 311]	24<br>[14.8, 33.6]	80.2<br>[64.1, 103]	-72.8<br>[-75.1, -68.9]	-45<br>[-46.2, -41.1]	27.6<br>[25.3, 30.4]	309<br>[247, 388]	-2.35<br>[-2.88, -1.64]
15	313<br>[215, 339]	16.6<br>[12.2, 25.3]	55.4<br>[52.3, 71.1]	-73.3<br>[-75.3, -71.7]	-45.7<br>[-47.4, -42.9]	27.6<br>[25.7, 29.9]	308<br>[226, 377]	-2.31<br>[-2.72, -1.65]
16	135<br>[105, 180]	7.96<br>[6.54, 10.2]	57.4<br>[50.5, 69]	-74.5<br>[-76.4, -72.5]	-46.3<br>[-48.4, -44.1]	27.8<br>[26.1, 30.6]	135<br>[109, 180]	-0.748<br>[-1.17, -0.516]

Table 4: **Summary characterization of GLIF parameters within clusters found by clustering GLIF4 parameters.** The single number in each cell is the median. The low and high quartiles are demarked in brackets below the median. Some neurons have the required stimuli for LIF models but do not have the stimuli required for higher level models. When there are less than five neurons that have the parameter, these values are denoted with a NaN.

	Q_2 (pC)	δt (ms)	slope	intercept (mV)	a_{spike} (V)	$1/(b_{spike})$ (s)	$(a_v)/(b_v)$	$log_{10}(b_v)$
	-2.16	4.43	0.243	18.6	0.00426	0.00191	0.25	2.02
1	[-2.34, -1.85]	[3.88, 4.66]	[0.189, 0.326]	[15.8, 20.4]	[0.00284, 0.00759]	[0.0011, 0.00577]	[0.213, 0.349]	[1.86, 2.1]
	-1.99	3.45	0.13	24	0.00271	0.00329	0.208	1.86
2	[-2.25, -1.72]	[3.1, 3.75]	[0.0727, 0.222]	[21.4, 25.3]	[0.000618, 0.00791]	[0.00102, 0.0113]	[0.14, 0.297]	[1.72, 2.1]
	-1.9	4.35	0.438	8.56	0.00222	0.00604	0.224	1.77
3	[-2.21, -1.62]	[3.7, 4.75]	[0.348, 0.519]	[7.37, 14]	[0.0017, 0.00325]	[0.00234, 0.0132]	[0.185, 0.27]	[1.64, 2.13]
	-1.51	2.8	0.419	13.5	0.00277	0.00683	0.181	1.75
4	[-2.04, -1.04]	[2.55, 3.6]	[0.21, 0.518]	[9.94, 19.1]	[0.000972, 0.00441]	[0.000997, 0.0211]	[0.114, 0.259]	[1.5, 2.22]
	-2.72	6.05	0.476	10.4	0.00251	0.00296	0.346	1.93
5	[-3.36, -2.28]	[4.95, 7.5]	[0.352, 0.591]	[5.3, 14]	[0.00129, 0.00437]	[0.000967, 0.00572]	[0.284, 0.388]	[1.79, 2.02]
	-1.56	4.55	0.655	2.17	0.00213	0.00733	0.396	1.81
6	[-2.12, -0.809]	[4.14, 7.35]	[0.534, 0.792]	[-1.28, 3.32]	[0.00126, 0.00346]	[0.0045, 0.0112]	[0.315, 0.43]	[1.52, 2]
	-2.67	3.6	0.705	-0.666	0.00415	0.00624	0.356	1.72
7	[-3.35, -0.877]	[3.1, 4.95]	[0.632, 0.853]	[-3.28, 5.67]	[0.00199, 0.00723]	[0.00252, 0.0149]	[0.285, 0.417]	[1.51, 1.87]
	-2.32	4.45	0.992	-7.82	0.00281	0.00293	0.284	1.68
8	[-2.77, -1.93]	[3.64, 5.55]	[0.847, 1.14]	[-12.1, -3.22]	[0.00185, 0.00608]	[0.00155, 0.00559]	[0.195, 0.378]	[1.56, 1.87]
	-1.34	4.83	1.17	-15	0.00211	0.013	0.234	1.48
9	[-2.11, -0.649]	[3.4, 7.18]	[1, 1.35]	[-21.3, -11.5]	[0.00182, 0.003]	[0.00787, 0.0302]	[0.15, 0.304]	[1.36, 1.66]
	-0.371	2.7	0.375	8.67	0.00209	0.046	0.134	1.42
10	[-0.57, 0.302]	[2.3, 3.05]	[0.242, 0.425]	[6.47, 11.3]	[0.00165, 0.00245]	[0.0293, 0.0913]	[0.105, 0.202]	[1.38, 1.58]
	-0.371	3.73	0.35	4.76	0.00254	0.037	0.249	1.37
11	[-0.741, 0.172]	[3.2, 4.3]	[0.188, 0.535]	[0.981, 7.57]	[0.00211, 0.00324]	[0.0323, 0.0418]	[0.147, 0.332]	[1.09, 1.82]
	1.12	2.2	0.511	-0.5	0.00192	0.0365	0.261	1.57
12	[0.658, 1.92]	[1.9, 2.8]	[0.382, 0.623]	[-3.91, 3.43]	[0.00132, 0.00287]	[0.0234, 0.0783]	[0.164, 0.316]	[1.34, 1.88]
	-0.594	3.92	0.469	8.61				
13	[-2.04, 0.895]	[3.35, 4.45]	[0.398, 0.524]	[5.92, 10.3]	NaN	NaN	NaN	NaN
	-1.53	4.15	0.198	15.6	0.00197	0.00402	0.196	1.86
14	[-1.81, -1.02]	[3.76, 6.17]	[0.0653, 0.313]	[11.9, 20]	[0.000571, 0.00336]	[0.00218, 0.00987]	[0.121, 0.321]	[1.72, 2.09]
	-0.899	4.1	0.62	4.57	0.00283	0.0194	0.238	1.87
15	[-1.36, -0.171]	[3.25, 4.79]	[0.491, 0.716]	[0.984, 7.1]	[0.00133, 0.0087]	[0.00248, 0.0587]	[0.211, 0.272]	[1.61, 2.17]
	0.161	1.7	0.146	11.1	0.00319	0.00378	0.228	2.18
16	[-0.377, 0.591]	[1.5, 1.9]	[0.0544, 0.263]	[7.84, 14.4]	[0.00162, 0.00565]	[0.00189, 0.00853]	[0.147, 0.287]	[1.94, 2.35]

Table 5: SECOND HALF OF TABLE 4

	$\tau_m (ms)$	R_i (MΩ)	V_{rest} (mV)	$I_{threshold}$† (pA)	$V_{threshold}$† (mV)	V_{peak}† (mV)	$V_{fasttrough}$† (mV)	V_{trough}†
	19.4	144	-76.8	130	-38	39.3	-48.3	-54.5
1	[17.4, 21.7]	[137, 157]	[-78.8, -75.4]	[110, 155]	[-40.1, -36.1]	[36.2, 42.9]	[-52, -47]	[-55.9, -53]
	17.9	154	-78.9	130	-36.7	38.7	-50	-54.8
2	[14.1, 20.5]	[126, 177]	[-81.2, -77.5]	[110, 190]	[-40.5, -35.8]	[35.2, 41]	[-53.4, -48]	[-56.5, -52.4]
	20.2	180	-74.3	90	-38.5	39.3	-49.3	-54.7
3	[16.9, 23.6]	[154, 198]	[-77.4, -73]	[70, 120]	[-41.3, -36.4]	[31.6, 42.8]	[-51.7, -47.2]	[-56.8, -53.5]
	16.4	164	-76.9	110	-36.5	33.2	-50.7	-55.8
4	[8.8, 20.7]	[112, 193]	[-80.5, -73.3]	[90, 190]	[-41, -34]	[21.5, 40.1]	[-54.5, -48.5]	[-56.6, -52.1]
	20.4	141	-76.1	130	-36.8	36.7	-47.2	-54.2
5	[17.3, 23.2]	[116, 171]	[-78.2, -74.1]	[90, 190]	[-38.4, -33.8]	[32.9, 39.8]	[-49.1, -45.3]	[-57, -52.3]
	19.1	141	-68.3	70	-39	38.7	-48.5	-55.3
6	[15.5, 26.6]	[117, 203]	[-70.8, -63.2]	[50, 128]	[-42.4, -35]	[35, 44.8]	[-50.5, -46.7]	[-57, -53.7]
	15.8	160	-74.5	150	-35.2	35.3	-47.9	-52.8
7	[14.2, 20.4]	[137, 194]	[-77.5, -71.4]	[90, 210]	[-38.7, -33.3]	[30.1, 40.9]	[-50.7, -46]	[-55.4, -48.5]
	14	126	-77.1	190	-36.4	34.4	-46.9	-52.7
8	[10.4, 19.3]	[94.5, 169]	[-78.6, -74.6]	[125, 250]	[-38.4, -34.3]	[24.1, 38.7]	[-49.1, -45.7]	[-54.5, -49.8]
	16.6	194	-69.5	90	-36	31.4	-48.2	-52.8
9	[11, 21.3]	[151, 225]	[-72.9, -66]	[65, 140]	[-40.8, -32.5]	[24.8, 38.5]	[-52.2, -45.4]	[-56.3, -48]
	12.6	271	-72	50	-42.1	28.9	-54	-55.8
10	[10.9, 15.8]	[236, 285]	[-73.2, -69.4]	[40, 70]	[-45.3, -38.5]	[21.4, 33.3]	[-57, -52.5]	[-57.4, -53.4]
	22.9	305	-65.1	30	-39.9	31.3	-51.9	-55.2
11	[15.1, 29.5]	[244, 378]	[-67.2, -62.8]	[22.5, 50]	[-43.1, -37.1]	[25.5, 36.5]	[-55.5, -50.8]	[-56.8, -53.1]
	19.9	204	-71.1	90	-42.3	27.5	-58.6	-58.7
12	[12.1, 25.8]	[167, 249]	[-73, -67]	[50, 120]	[-44.5, -39.4]	[21.4, 34.1]	[-61.6, -55.7]	[-61.7, -55.8]
	17.9	153	-69.3	85	-40.9	35	-49.1	-55.2
13	[15.7, 25.2]	[119, 199]	[-70.9, -66.5]	[70, 115]	[-41.5, -39.6]	[30.3, 36.8]	[-52.5, -46.9]	[-56.6, -51.7]
	21.9	210	-72.3	70	-36.9	37.3	-50.2	-56.1
14	[13, 26.8]	[173, 239]	[-75.3, -68.5]	[57.5, 90]	[-38.9, -35.4]	[29.1, 43.3]	[-52, -48]	[-57.5, -53.7]
	12.8	197	-73.2	90	-38.5	29.8	-50.3	-51.8
15	[10.4, 18.5]	[171, 233]	[-75.1, -71.8]	[55, 127]	[-41.2, -35.9]	[21.7, 38]	[-52.4, -48.4]	[-54.3, -50.4]
	7.1	108	-74.5	250	-38	14.7	-61.3	-61.4
16	[6.2, 8.31]	[83.3, 142]	[-76.4, -72.5]	[170, 320]	[-41.3, -34.8]	[11.6, 20]	[-64.3, -57.8]	[-64.4, -58]

Table 6: **Data features of clusters found by clustering $GLIF_4$ parameters**. Information on the calculation of these parameters can be found in the Electrophysiological Features section of the Methods in the main text and at [1]. Abbreviations: * short square, † long square.

	up:downstroke†	up:downstroke*	sag (mV)	Avg. ISI (ms)	f-I curve slope (spikes $s^{-1}pA^{-1}$)	latency (s)	Adaptation	Max. burst index
1	3.82<br>[3.46, 4.04]	3.98<br>[3.77, 4.27]	0.0352<br>[0.0212, 0.0542]	89.5<br>[65.4, 104]	0.118<br>[0.0942, 0.169]	0.0521<br>[0.0358, 0.0618]	0.097<br>[0.0593, 0.157]	0.157<br>[0, 0.202]
2	3.59<br>[3.26, 3.72]	3.69<br>[3.16, 4.03]	0.0305<br>[0.018, 0.0474]	68.7<br>[54.4, 79.2]	0.157<br>[0.112, 0.192]	0.0412<br>[0.0379, 0.0529]	0.0505<br>[0.0185, 0.123]	0.229<br>[0.182, 0.302]
3	3.69<br>[3.13, 4]	3.74<br>[3.36, 4.26]	0.029<br>[0.0205, 0.0494]	72.3<br>[58.8, 90.2]	0.216<br>[0.158, 0.253]	0.0436<br>[0.0358, 0.061]	0.0398<br>[0.0239, 0.0733]	0.169<br>[0, 0.19]
4	3.34<br>[2.95, 3.54]	2.86<br>[2.51, 3.41]	0.0325<br>[0.022, 0.069]	74.6<br>[60.2, 86.3]	0.196<br>[0.141, 0.225]	0.0438<br>[0.0307, 0.0458]	0.0318<br>[0.00622, 0.0437]	0.166<br>[0, 0.285]
5	3.8<br>[3.41, 4.09]	3.95<br>[3.49, 4.19]	0.0596<br>[0.0384, 0.0841]	87.5<br>[75.4, 114]	0.123<br>[0.0901, 0.167]	0.0571<br>[0.0457, 0.0736]	0.0348<br>[0.0198, 0.0846]	0<br>[0, 0.156]
6	3.15<br>[2.75, 3.71]	3.28<br>[2.95, 3.94]	0.0851<br>[0.0436, 0.167]	77<br>[62, 94.5]	0.191<br>[0.171, 0.217]	0.0436<br>[0.028, 0.0517]	0.0296<br>[0.0135, 0.0543]	0.155<br>[0, 0.3]
7	2.58<br>[2.28, 2.94]	2.87<br>[2.54, 3.16]	0.124<br>[0.0955, 0.166]	76.5<br>[58.6, 93.5]	0.15<br>[0.0493, 0.173]	0.0325<br>[0.0244, 0.0457]	0.0372<br>[0.0167, 0.137]	0.212<br>[0, 0.285]
8	3.41<br>[2.94, 3.89]	3.53<br>[2.71, 3.93]	0.0436<br>[0.023, 0.101]	82.9<br>[69.3, 109]	0.181<br>[0.133, 0.21]	0.0462<br>[0.0334, 0.0649]	0.035<br>[0.016, 0.0539]	0.121<br>[0, 0.262]
9	2.87<br>[2.47, 3.62]	2.68<br>[2.38, 3.78]	0.0743<br>[0.0338, 0.129]	58.9<br>[43.6, 75.8]	0.238<br>[0.203, 0.306]	0.0366<br>[0.023, 0.0531]	0.0158<br>[0.00834, 0.0335]	0<br>[0, 0.0994]
10	2.78<br>[1.78, 2.97]	2.79<br>[1.89, 2.94]	0.101<br>[0.0829, 0.13]	30.8<br>[23, 43.5]	0.379<br>[0.164, 0.547]	0.0141<br>[0.0125, 0.0196]	0.021<br>[0.0127, 0.0448]	0<br>[0, 0.106]
11	2.58<br>[2.21, 3.31]	2.52<br>[2.18, 2.96]	0.0499<br>[0.0301, 0.0781]	40.7<br>[33.5, 56.7]	0.283<br>[0.138, 0.378]	0.018<br>[0.0134, 0.0305]	0.0158<br>[0.0102, 0.0377]	0<br>[0, 0.141]
12	1.77<br>[1.58, 2.08]	1.82<br>[1.63, 2.07]	0.0733<br>[0.0287, 0.12]	39.5<br>[28.3, 57.5]	0.334<br>[0.17, 0.434]	0.0233<br>[0.0182, 0.0332]	0.032<br>[0.0128, 0.151]	0<br>[0, 0]
13	2.86<br>[2.68, 3.11]	2.98<br>[2.84, 3.12]	0.133<br>[0.0856, 0.154]	77<br>[63.5, 81]	0.162<br>[0.0366, 0.2]	0.0325<br>[0.0248, 0.0545]	0.042<br>[0.0165, 0.0566]	0.315<br>[0.169, 0.442]
14	3.27<br>[2.98, 3.7]	3.23<br>[2.84, 3.85]	0.0453<br>[0.0266, 0.0784]	61.4<br>[49.7, 81.2]	0.203<br>[0.173, 0.273]	0.0436<br>[0.0343, 0.0584]	0.0184<br>[0.00482, 0.0299]	0.148<br>[0, 0.215]
15	2.9<br>[2.53, 3.25]	2.88<br>[2.54, 3.29]	0.055<br>[0.0389, 0.0792]	57.7<br>[48.2, 89.1]	0.188<br>[0.132, 0.237]	0.016<br>[0.0114, 0.0383]	0.023<br>[0.0119, 0.0653]	0.101<br>[0, 0.245]
16	1.3<br>[1.2, 1.41]	1.41<br>[1.34, 1.57]	0.0466<br>[0.0314, 0.0673]	14.3<br>[10.9, 20.5]	0.782<br>[0.618, 1]	0.0109<br>[0.00814, 0.0162]	0.00365<br>[0.00159, 0.0275]	0<br>[0, 0]

Table 7: **Second half of Table 6**. Note that average ISI and adaptation is not included in the clustering because these values were not available for all neurons.

Supplementary Methods

Model Definitions

The five different GLIF models are described below, in order of increasing level of complexity, with their evolution equations and reset rules. For all the models, C represents the membrane capacitance, R, is the membrane resistance, E_L is the resting potential and $I_e(t)$ is an external current injected into the cell. t_+ and t_- represent the time just after and before a spike respectively.

Leaky Integrate-And-Fire ($GLIF_1$)

The traditional Leaky Integrate-And-Fire (LIF) neuron is a hybrid system characterized by an evolution equation for the membrane potential V(t). We refer to this model as $GLIF_1$, and it is the starting point for further GLIF models.

$$V'(t) = \frac{1}{C}\Big(I_e(t) - \frac{1}{R}\left(V(t) - E_L\right)\Big) \tag{1}$$

When the membrane potential becomes larger than a threshold, $V(t) > \Theta$, (which in this case is just the instantaneous threshold, Θ_∞), a reset rule is evoked which sets the membrane voltage to a reset voltage, V_r.

$$V(t) \leftarrow V_r \tag{2}$$

Leaky Integrate-And-Fire with biologically defined reset rules ($GLIF_2$)

$GLIF_2$ has an evolution equation for two state variables, the membrane potential $V(t)$ and a spike-dependent threshold component $\Theta_s(t)$ which is updated by spikes and decays back to zero with a time constant $(1/b_s)$.

$$\begin{aligned} V'(t) &= \frac{1}{C}\Big(I_e(t) - \frac{1}{R}\left(V(t) - E_L\right)\Big) \\ \Theta_s'(t) &= -b_s\Theta_s(t) \end{aligned} \tag{3}$$

along with a reset rule for both voltage and the spike-dependent threshold if the membrane potential becomes larger than a threshold $V(t) > \Theta_\infty + \Theta_s(t)$

$$\begin{aligned} V(t_+) &\leftarrow E_L + f_v \times \left(V(t_-) - E_L\right) - \delta V \\ \Theta_s(t_+) &\leftarrow \Theta_s(t_-) + \delta\Theta_s \end{aligned} \tag{4}$$

The update to the spike-dependent component of the threshold is additive, with $\delta\Theta_s$ added after every spike. The update to the membrane potential has a multiplicative coefficient, f_v, and an additive constant, δV

Leaky Integrate-And-Fire with after-spike currents ($GLIF_3$)

In GLIF models, the rapid membrane potential fluctuation due to the fast voltage-activated (i.e. sodium and potassium) ion currents during a spike, is considered separately from the slow, subthreshold region of the membrane potential and is incorporated into the reset rules. However, ion currents activated by a spike could have effects over longer time scales. After the stereotypical, sharp membrane potential transitions during an action potential are eliminated, the longer term effects are modeled as additional currents I_j with pre-defined time constants $(1/k_j)$,

$$\begin{aligned} I_j'(t) &= -k_j I_j(t);\ j = 1, ..., N \\ V'(t) &= \frac{1}{C}\Big(I_e(t) + \sum_j I_j(t) - \frac{1}{R_{ASC}}\left(V(t) - E_L\right)\Big) \end{aligned} \tag{5}$$

Here R_{ASC} is used to explicitly point out that the resistance is fit along with the after-spike currents. The update rule, which applies if $V(t) > \Theta_\infty$, is given by:

$$\begin{aligned} I_j(t_+) &\leftarrow f_j \times I_j(t_-) + \delta I_j \\ V(t_+) &\leftarrow V_r \end{aligned} \tag{6}$$

with the currents updated using a multiplicative constant $f_j = \exp(-k_j \delta t)$ (because after-spike currents decay through the spike cut length) and an additive constant, δI_j.

Leaky Integrate-And-Fire with biologically defined reset rules and after-spike currents ($GLIF_4$)

Combining the biological reset rules model, $GLIF_2$, with the after-spike current model, $GLIF_3$, described above gives a model defined by:

$$\begin{aligned} I_j'(t) &= -k_j I_j(t);\ j = 1, ..., N \\ V'(t) &= \frac{1}{C}\left(I_e(t) + \sum_j I_j(t) - \frac{1}{R_{ASC}}(V(t) - E_L)\right) \\ \Theta_s'(t) &= -b_s \Theta_s(t) \end{aligned} \tag{7}$$

$$\tag{8}$$

The update rule, which applies if $V(t) > \Theta_s(t) + \Theta_\infty$,

$$\begin{aligned} I_j(t_+) &\leftarrow f_j \times I_j(t_-) + \delta I_j \\ V(t_+) &\leftarrow E_L + f_v \times (V(t_-) - E_L) - \delta V \\ \Theta_s(t_+) &\leftarrow \Theta_s(t_-) + \delta \Theta_s \end{aligned} \tag{9}$$

$$\tag{10}$$

Leaky Integrate-And-Fire with biologically defined reset rules, after-spike currents, and a voltage dependent threshold ($GLIF_5$)

For the $GLIF_5$ model, the state variables are the membrane potential, $V(t)$ (as in all GLIF models), a threshold component which is evoked when there is a spike, Θ_s (as introduced in $GLIF_2$), a set of after-spike currents $I_j(t)$ (as introduced in $GLIF_3$), and a new additional threshold parameter that is dependent of the membrane potential (Θ_v). It is assumed that these state variables evolve in a linear manner between spikes:

$$\begin{aligned} I_j'(t) &= -k_j I_j(t);\ j = 1, ..., N \\ V'(t) &= \frac{1}{C}\left(I_e(t) + \sum_j I_j(t) - \frac{1}{R_{ASC}}(V(t) - E_L)\right) \\ \Theta_s'(t) &= -b_s \Theta_s(t) \\ \Theta_v'(t) &= a_v(V(t) - E_L) - b_v \Theta_v(t) \end{aligned} \tag{11}$$

Where $1/b_v$ is the time constant of the voltage-dependent component of the threshold and, a can be interpreted as a 'leak-conductance' for the voltage-dependent component of the threshold.
If $V(t) > \Theta_v(t) + \Theta_s(t) + \Theta_\infty$, a spike is generated and the state variables are updated:

$$
\begin{aligned}
I_j(t_+) &\leftarrow f_j \times I_j(t_-) + \delta I_j \\
V(t_+) &\leftarrow E_L + f_v \times (V(t_-) - E_L) - \delta V \\
\Theta_s(t_+) &\leftarrow \Theta_s(t_-) + \delta\Theta_s \\
\Theta_v(t_+) &\leftarrow \Theta_v(t_-)
\end{aligned} \tag{12}
$$

For all state variables, $X_k(t)$, the value immediately after the spike, $X_k(t_+)$ was related to the value immediately before the spike, $X_k(t_-)$, via a set of update parameters: f_k represented the fraction of the prespike value of X_k which is maintained after the spike and δX_k represents the values updated by a spike.

Parameter fitting and distributions

Model parameters are extracted from the electrophysiology data as described below. Python code for all algorithms can be found in the Allen Software Development Kit (SDK) on the Allen Cell Types Database website. All code was executed using Python 2.7. In Figures 7,1, 2, and 3 we show slices of the parameter space obtained via fitting.

Parameter descriptions

Spike initiation time and threshold [t_s, Θ_{bio}]: Spikes are typically detected by the following procedure. The upstroke of a spike is defined as the portion of the action potential wave form between the instant at which the rise in potential for a given time exceeds 20 mV/ms and the peak of the action potential. For each neuron, the average value of the maximum dV/dt during the upstrokes of all action potentials is calculated. The time of spike initiation and threshold is then defined by the time and voltage at which dV/dt reaches 5% of the average maximum dV/dt during an upstroke.

For stimuli that involve a short, intense current pulse stimulation, the passive rise in voltage preceding a spike can exceed the standard 20 mV/ms initial detection value, leading to inaccurate spike time identification. In those cases, the maximum dV/dt is measured during the current pulse, and the spike detection value is adjusted to be 10% higher than that. In addition, the threshold identification value (typically 5% of the average maximum dV/dt) is also adjusted to ensure that it is at least 20% higher than the dV/dt at the end of the stimulus pulse.

Spike cut length and voltage reset [δt, V_{t+}]: GLIF models aim to reproduce the timing of the spikes via sub-threshold data (not the voltage waveform dictated by the highly non-linear ion fluctuations during the action potential). Therefore, the action potential waveforms are removed from the voltage trace. Here we use a principled way to estimate the duration of the spike that is removed and the reset voltage in models where biological reset rules are implemented ($GLIF_2$, $GLIF_4$, and $GLIF_5$). Action potentials in a neuron have stereotyped shapes with a sharp rise, followed by a hyperpolarized refractory period and a return to a voltage that is most often lower than the voltage before the spike (voltage reset). As shown in Supplementary Figure 1, we align all of the action potentials of the training noise data (noise 1) to the spike initiation and ask at what time within a window of 1 to 10 ms after spike initiation does the post spike voltage best predict the voltage at spike initiation given a linear dependence. i.e. a line is fit between the voltages at spike initiation (prespike voltages) and the voltages at each time point after spike initiation (postspike voltages). The fit which minimizes the residuals is chosen to define the time at which the spike ends. In all models, the duration of the spike is removed from the voltage traces. For the $GLIF_2$, $GLIF_4$, and $GLIF_5$ models, the postspike voltage is reset via the best fit linear model $V(t+) = E_L + f_v * (V(t-) - E_L) + \delta V$, (main article equation 5, supplementary equations 4, 9, and 12) where $V(t-)$ is the voltage of the model before the spike, δV is the voltage intercept, f_v is the slope of the fit, and $V(t+)$ is the voltage of the model after the spike.

Instantaneous threshold [Θ_∞]: All models (neurons) spike when the voltage crosses threshold. An estimate of the instantaneous threshold, Θ_∞, is the voltage at spike initiation of the lowest amplitude supra-threshold short square (Figure 2 of the main text). Threshold is a critical parameter affecting the general excitability of the cell. The relationship between voltage and stimulus likely becomes non-linear near threshold. Because small errors in the Θ_∞ measurement will greatly influence spiking behavior Θ_∞ is tuned during the post-hoc optimization of every model such that the likelihood of the observed spike train in the training stimulus is maximized.

Resting potential [E_L]: The resting potential was defined as the mean resting potential of the noise 1 sweeps calculated by averaging the pre-stimulus membrane potential.

Resistance [R], Capacitance[C], and after-spike currents [$I_j(t)$]: Because the GLIF model is linear, many of the parameters can be fit via linear regression on subthreshold data similar to the methods in [2]. Initially, R and C are calculated via a linear regression of the subthreshold noise in the first epoch of noise stimulation within the three-epoch noise sweeps (this stimulus does not elicit any spikes) as observed in Figure 2d. The neurons which elicited spikes during the subthreshold stimulation were eliminated from subsequent fits. Please see section 12 for example calculations. These values of resistance are used for models that do not include after-spike currents ($GLIF_1$, $GLIF_2$).

For the GLIF model levels where after-spike currents are included ($GLIF_3$, $GLIF_4$, $GLIF_5$), after-spike currents and resistance are fit via a linear regression on the periods between the spikes in the supra-threshold noise data. During this calculation, the capacitance and resting potential are forced to the previously calculated values. We choose to force capacitance while continuing to fit resistance because capacitance is a consistent property of the membrane which does not change based on the state of a neuron. However, the resistance of membrane will depend on the state of a neuron and will most likely differ with the activation of ion currents whose subthreshold affects are modeled here with after-spike currents.

After-spike currents are modeled using exponential decaying basis functions with an amplitude and a time scale (the dynamics of the exponential functions are described in equation 7). The time scales and amplitudes are obtained by providing two from a set of five basis currents with varied time scales ($1/k_j$) ([3.33, 10, 33.3, 100, 333.33] ms) to a generalized linear model (GLM). The GLM (implemented via Python's statsmodels.api.GLM) is used to calculate the amplitudes corresponding to each basis current and the resistance by regressing the sum of the derivative of the voltage and the external current divided by the capacitance ($\frac{dV}{dt} + \frac{I_e}{C}$) against the basis currents and the leak term. C and E_L are calculated as mentioned in the above text. The GLM is run with all combinations of two of the five possible time scales leading to a choice among ($^5C_2 = 10$) pairs of basis currents. The optimal pair of basis currents that yield the maximum log-likelihood value are chosen. The total charge, Q, deposited by one after-spike current in the neuron per spike is $\delta I_j/k_j$. Since total charge is a biologically relevant parameter and it is easier to perform clustering on continuous values, we often use the total charge (Table 2 of the main article) instead of the individual parameters, δI_j, and, k_j (where k_j are discrete values and the best two out of 5 possible values are chosen; see above), represented in equations 3 and 7 of the main article and supplementary equations 5, 6, 7, 9, 11 and 12, Figure 3 of the main article and Supplementary Figure 2, and Supplementary Tables 2 and 4.

Spike initiated component of the threshold [Θ_s]: The evolution of the threshold of $GLIF_2$, $GLIF_4$, and $GLIF_5$ contains a spiking component of the threshold, Θ_s . This contribution to the threshold represents the effect spiking has on the threshold of the neuron due to the inactivation of a voltage-dependent sodium current. This inactivation can be interpreted as a rise in the threshold of the neuron, and the movement from the inactive to a closed state can be modeled as a linear dynamical process. The change in threshold is fit with an exponential, and the values of its amplitude and time constant are calculated from the triple short square sweep set data as seen in Figure 3. For each pulse in the triple short square data that produces a spike, the voltage at spike initiation (the threshold) is calculated. The mean of the threshold of the first spike of each individual triple short square stimulus was taken to be the reference threshold. For all spikes that are not first spikes in each individual triple stimulus, the time since the last spike is calculated (referred

to as an ISI) along with the threshold relative to the reference threshold. An exponential is then fit to the ISI versus threshold data. The exponential is forced to decay to the reference threshold.

Subthreshold voltage dependent component of the threshold [Θ_v]: $GLIF_5$ contains both a spiking component of the threshold, Θ_s, (see above) and a subthreshold voltage component of the threshold, Θ_v. The subthreshold voltage component represents the effect of subthreshold membrane potential on the threshold (e.g., voltage dependence of sodium current reactivation). The voltage component of the threshold evolves according to $\Theta_v'(t)$ in equation 4 of the main text and supplementary equation 11. In order to fit a_v and b_v, the sum of the squared error between the voltage component of the threshold of the biological neuron Θ_{vbio} and the voltage component of the threshold of the model Θ_v is minimized. Θ_{vbio} is found by subtracting the spike component of the threshold, Θ_s, from the actual value of the voltage at the time of spike initiation, Θ_{bio} ($\Theta_{vbio} = \Theta_{bio} - \Theta_s$). Although one could simulate the voltage component of the threshold using Euler's method, the analytical solution (for derivation please see Supplementary Methods section "Analytical solution for the dynamics of the voltage component of the threshold") was used to speed up the minimization process. Minimization was preformed using a Nelder-Mead simplex algorithm (fmin in the Python scipy optimization tool box: scipy.optimize.fmin).

Using linear regression to solve for variables using a noisy stimulus

One way to fit the resistance, R, capacitance, C, and resting potential, E_L, is to use linear regression on the voltage, V, in response to a noisy stimulus, I. The standard leaky integrate and fire model for the dynamics of a neuron is:

$$C\frac{dV}{dt} = -\frac{(V - E_L)}{R} + I \tag{13}$$

This equation can be rearranged so that it fits into the form $y = X\beta + \epsilon$ where y is the dependent variable, X is the independent variable and β are the parameters being fit. For implementation this means that the parameters being fit should be associated with the matrix on the (rhs) of the equation, while the dependent variable and the known parameters should be on the left hand side (lhs) of the equation. After the equation has been arranged in the proper form, a standard least squares algorithm such as *np.linalg.lstsq* in python can be used.

For example let us say we want to fit E_L and R in Equation 13. Equation 13 can be rearranged as follows:

$$\begin{aligned}
\frac{dV}{dt} &= \frac{-V + E_L}{RC} + \frac{I}{C} \\
\frac{\triangle V}{\triangle t} &= \frac{-V}{RC} + \frac{E_L}{RC} + \frac{I}{C} \\
V_{t+1} - V_t &= \frac{-V_t \triangle t}{RC} + \frac{E_L \triangle t}{RC} + \frac{I_t \triangle t}{C} \\
V_{t+1} &= \frac{-V_t \triangle t}{RC} + V_t + \frac{E_L \triangle t}{RC} + \frac{I_t \triangle t}{C} \\
&= V_t(1 - \frac{\triangle t}{RC}) + \frac{E_L \triangle t}{RC} + \frac{I_t \triangle t}{C}
\end{aligned} \tag{14}$$

Here, $\triangle t$ is the amount of time between V_t and V_{t+1} (time step) where t is the timestep index which runs from $t = 1$ to $t = T$ where T is the total number of time steps in the data.

In vector form, Equation 14 is:

$$\begin{bmatrix} V_2 \\ V_3 \\ . \\ . \\ V_T \end{bmatrix} = \begin{bmatrix} V_1 \\ V_2 \\ . \\ . \\ V_{T-1} \end{bmatrix} \left(1 - \frac{\triangle t}{RC}\right) + \begin{bmatrix} 1 \\ 1 \\ . \\ . \\ 1 \end{bmatrix} \frac{E_L \triangle t}{RC} + \begin{bmatrix} I_1 \\ I_2 \\ . \\ . \\ I_{T-1} \end{bmatrix} \frac{\triangle t}{C}$$

Fitting R, C, and E_L results in the following equation:

$$\begin{bmatrix} V_2 \\ V_3 \\ . \\ . \\ V_N \end{bmatrix} = \begin{bmatrix} V_1 & 1 & I_1 \\ V_2 & 1 & I_2 \\ . & . & . \\ . & . & . \\ V_{N-1} & 1 & I_{N-1} \end{bmatrix} \begin{bmatrix} \left(1 - \frac{\triangle t}{RC}\right) \\ \\ \frac{E_L \triangle t}{RC} \\ \\ \frac{\triangle t}{C} \end{bmatrix}$$

The solution is a vector *out* with components,

$$\begin{aligned} out[1] &= \left(1 - \frac{\triangle t}{RC}\right) \\ out[2] &= \frac{E_L \triangle t}{RC} \\ out[3] &= \frac{\triangle t}{C} \end{aligned} \tag{15}$$

Solving this system of equations yields:

$$C = \frac{\triangle t}{out[3]} \tag{16}$$

$$R = \frac{- \triangle t}{C(out[1] - 1)} = \frac{- \triangle t}{\frac{\triangle t}{out[3]}(out[1] - 1)} = \frac{-out[3]}{(out[1] - 1)} \tag{17}$$

$$E_L = out[2]\left(\frac{RC}{\triangle t}\right) = out[2]\left(\frac{R\frac{\triangle t}{out[3]}}{\triangle t}\right) = out[2]\left(\frac{R}{out[3]}\right) \tag{18}$$

Now let's say that we do not want to fit the capacitance during the regression. In this case the term containing the I vector will be shifted to the left hand side of the equation:

$$\begin{bmatrix} V_2 \\ V_3 \\ . \\ . \\ V_T \end{bmatrix} - \begin{bmatrix} I_1 \\ I_2 \\ . \\ . \\ I_{T-1} \end{bmatrix} \frac{\triangle t}{C} = \begin{bmatrix} V_1 \\ V_2 \\ . \\ . \\ V_{T-1} \end{bmatrix} \left(1 - \frac{\triangle t}{RC}\right) + \begin{bmatrix} 1 \\ 1 \\ . \\ . \\ 1 \end{bmatrix} \frac{E_L \triangle t}{RC}$$

In matrix form:

$$\begin{bmatrix} V_2 - I_1 \frac{\triangle t}{C} \\ V_3 - I_2 \frac{\triangle t}{C} \\ . \\ . \\ V_T - I_{T-1} \frac{\triangle t}{C} \end{bmatrix} = \begin{bmatrix} V_1 & 1 \\ V_2 & 1 \\ . & . \\ . & . \\ V_{T-1} & 1 \end{bmatrix} \begin{bmatrix} \left(1 - \frac{\triangle t}{RC}\right) \\ \\ \frac{E_L \triangle t}{RC} \end{bmatrix}$$

Solving this system of equations yields:

$$R = \frac{- \triangle t}{C(out[1] - 1)} \tag{19}$$

$$E_L = \left(\frac{RC}{\triangle t}\right) out[2] \tag{20}$$

Above, we solved this problem by separating $\triangle V$ into the voltages before and after the time step $\triangle t$, i.e. $V_{t+1} - V_t$. This problem can also be solved by calculating $\frac{\triangle V}{\triangle t}$ directly from the data.

Analytical solution for the dynamics of the voltage component of the threshold

The voltage component of the threshold evolves according to the following dynamics:

$$\frac{d\theta}{dt} = a(V(t) - E_L) - b(\theta(t) - \theta_\infty) \tag{21}$$

where θ is the voltage component of the threshold, V is the voltage of the neuron, E_L is the resting potential, θ_∞, and a and b are constants which will be fit to the data.
For completeness, below we will outline the analytical solution to equation (21).

The solution to a differential equation with the form:

$$\dot{x} + p(t)x = q(t) \tag{22}$$

is

$$x(t) = \frac{1}{u(t)} \int u(t)q(t)dt + K \tag{23}$$

where K is the standard integration constant and

$$u(t) = e^{\int p(t)dt} \tag{24}$$

Equation (21) can be rearranged into this form:

$$\frac{d\theta}{dt} + b\theta = a(V(t) - E_L) + b\theta_\infty \tag{25}$$

From equations (22), (24) and (25),

$$\begin{aligned} u(t) &= e^{\int b dt} = e^{bt} \\ q(t) &= a(V(t) - E_L) + b\theta_\infty \end{aligned} \tag{26}$$

Therefore:

$$\begin{aligned} \theta(t) &= \frac{1}{e^{bt}} \int e^{bt}[a(V(t) - E_L) + b\theta_\infty]dt + K \\ &= \frac{1}{e^{bt}} \int [ae^{bt}V(t) - ae^{bt}E_L + b\theta_\infty e^{bt}]dt + K \end{aligned} \tag{27}$$

Similarly, it can be shown that for a constant current I with $V(t = 0) = V_0$ and $g = \frac{1}{R}$, the analytical solution for V(t) is,

$$V(t) = V_0 e^{-\frac{tg}{c}} + \left(\frac{I + gE_L}{g}\right)(1 - e^{-tg/c}) \tag{28}$$

Plugging in $V(t)$ in equation (27) and defining $\beta = \frac{I+gE_L}{g}$ gives:

$$\begin{aligned} \theta(t) &= \frac{1}{e^{bt}} \int [ae^{bt}(V_0 e^{-\frac{tg}{c}} + \beta(1 - e^{-tg/c})) - ae^{bt}E_L + b\theta_\infty e^{bt}]dt + K \\ &= \frac{1}{e^{bt}} \int [ae^{bt}V_0 e^{-\frac{tg}{c}} + ae^{bt}\beta(1 - e^{-tg/c}) - ae^{bt}E_L + b\theta_\infty e^{bt}]dt + K \\ &= \frac{1}{e^{bt}} \int [aV_0 e^{(b-\frac{g}{c})t} + a\beta e^{bt} - a\beta e^{(b-\frac{g}{c})t} - ae^{bt}E_L + b\theta_\infty e^{bt}]dt + K \end{aligned} \tag{29}$$

Setting $\alpha = (b - \frac{g}{c})$

$$\begin{aligned}\theta(t) &= \frac{1}{e^{bt}}\left[\frac{aV_0}{\alpha}e^{\alpha t} + \frac{a\beta}{b}e^{bt} - \frac{a\beta}{\alpha}e^{\alpha t} - \frac{aE_L}{b}e^{bt} + \frac{b\theta_\infty}{b}e^{bt} + K\right] \\ &= \frac{aV_0}{\alpha}e^{-\frac{g}{c}t} + \frac{a\beta}{b} - \frac{a\beta}{\alpha}e^{-\frac{g}{c}t} - \frac{aE_L}{b} + \theta_\infty + \frac{K}{e^{bt}} \\ &= \left[\frac{aV_0}{\alpha} - \frac{a\beta}{\alpha}\right]e^{-\frac{g}{c}t} + \frac{a\beta}{b} - \frac{aE_L}{b} + \theta_\infty + \frac{K}{e^{bt}} \\ &= \frac{a}{\alpha}(V_0 - \beta)e^{-\frac{g}{c}t} + \frac{K}{e^{bt}} + \frac{a}{b}(\beta - E_L) + \theta_\infty \end{aligned} \tag{30}$$

at $t = 0$, $\theta(t) = \theta_0$. Therefore:

$$\theta_0 = \frac{a}{\alpha}(V_0 - \beta) + K + \frac{a}{b}(\beta - E_L) + \theta_\infty \tag{31}$$

$$K = \theta_0 - \frac{a}{\alpha}(V_0 - \beta) - \frac{a}{b}(\beta - E_L) - \theta_\infty \tag{32}$$

Plugging in K in the last line of equation (30):

$$\theta(t) = \frac{a}{\alpha}(V_0 - \beta)e^{-\frac{g}{c}t} + \frac{1}{e^{bt}}\left[\theta_0 - \frac{a}{\alpha}(V_0 - \beta) - \frac{a}{b}(\beta - E_L) - \theta_\infty\right] + \frac{a}{b}(\beta - E_L) + \theta_\infty \tag{or}$$

$$\begin{aligned} &= \frac{a}{b - \frac{g}{c}}\left(V_0 - \frac{I + gE_L}{g}\right)e^{-\frac{g}{c}t} \\ &\quad + \frac{1}{e^{bt}}\left[\theta_0 - \frac{a}{b - \frac{g}{c}}\left(V_0 - \frac{I + gE_L}{g}\right) - \frac{a}{b}\left(\frac{I + gE_L}{g} - E_L\right) - \theta_\infty\right] \\ &\quad + \frac{a}{b}\left(\frac{I + gE_L}{g} - E_L\right) + \theta_\infty \end{aligned} \tag{33}$$

Post-Hoc Optimization

An additional non-linear optimization step was performed to optimize the instantaneous threshold, θ_∞, using a Nelder-Mead simplex algorithm (Python scipy module scipy.optimize.fmin). The instantaneous threshold is an essential parameter governing the excitability of the neuron. As the current/voltage dependence for the neuron becomes highly nonlinear near the threshold, and we rely on linear models. To better fit the excitability of the models we search for the value of the instantaneous threshold which best predicts the spike train during the test stimulus. Comparing Figures 6 and 8 of the Supplementary Material illustrates the importance of the post-hoc optimization on explained variance.

Forced-spike Model Paradigm

During optimization, each spike should be fit without erroneous after-spike current history affecting the fitting of the subsequent spikes. The model voltage, threshold, and after-spike amplitudes are forced to reset at the time the biological neuron spikes. The reset rules are denoted in the GLIF model section above. When a model is run in its normal forward-running manner, the prespike values of the voltage, $V(t_-)$, threshold, $\Theta(t_-)$, and after-spike currents, $I_j(t_-)$, are the values of the model at the point where the model voltage crosses the model threshold. In the forced spike paradigm, to ensure that the voltage of the model is reset below the threshold of the model, the prespike voltage was set equal to the prespike threshold, $V(t_-) = \Theta(t_-)$ at the time of the neuron spike. Note that this reset only affects $GLIF_2$, $GLIF_4$, $GLIF_5$, where the prespike voltage affects the postspike voltage (Equation 5 of the main article and Supplementary Equations 4, 9, and 12).

Objective Function: Maximum Likelihood Based on Internal Noise (MLIN)

Similar to a series of previous studies [3, 4, 5, 2], the likelihood that the observed spike train was obtained by the model was maximized by minimizing the negative log-likelihood. However, the exact method of constructing the likelihood was different in that the noise is not tuned but rather uses a direct estimate of a biological neurons internal noise (Maximum Likelihood based on Internal Noise).

Because these GLIF models are deterministic, estimating the likelihood required adding a source of noise external to the model. Rather than searching for the parameters of this noise, a parametric description of the internal noise of each neuron is used (Supplementary Figure 5). As this internal noise could depend on the membrane potential, and the most relevant potential is near threshold, the variation in membrane potential during the steady state period of the largest subthreshold square pulse response is characterized.

The probability density of the neuron being at a potential, v, away from its mean is fit with an exponentially decaying function (Supplementary Figure 5):

$$p(v) = \frac{1}{2\delta v} exp(-\frac{|v|}{\delta v}) \tag{34}$$

This variation was considered to be additive to the membrane potential of the deterministic neuron. This allowed the computation of the probability that a neuron with the given noise would produce a spike if the deterministic model had a difference between threshold and membrane potential:

$$\Delta V(t) = \Theta(t) - V(t) = \Theta_s(t) + \Theta_v(t) + \Theta_\infty - V(t) \tag{35}$$

$$p_{spike}(t) = \int_{\Delta V(t)}^{\infty} p(v)dv = \int_{\Delta V(t)}^{\infty} \frac{1}{2\delta v} exp(-\frac{|v|}{\delta v})dv = 1 - c(\Delta V(t)) \tag{36}$$

where c represents the cumulative distribution of the intrinsic noise, (Supplementary Figure 5, center panel).

The likelihood of the model producing a set of spikes at the times the biological neuron produces them is:

$$p_{spikes} = \prod_{t \in t_s} p_{spike}(t) \tag{37}$$

However, the model neuron must both produce spikes when the biological neuron does, and not produce spikes when the biological neuron does not.

The likelihood of a model neuron not producing spikes is not independent for two nearby time points, as the intrinsic noise has a nontrivial autocorrelation. To estimate the likelihood of the model neuron not producing spikes at the times the biological neuron does not produce spikes, one sample for the internal noise is drawn for each time period of the autocorrelation, and following this time scale a new independent sample is drawn. As such, the inter-spike intervals are binned with bin sizes equal to the autocorrelation (typical autocorrelation time scales are much smaller than the inter-spike intervals). The grid times start from a spike time and advance by the autocorrelation time scale, ending at a predefined short time (5 ms) before the next spike. To estimate the likelihood of a neuron not producing a spike within a bin, the minimal difference between threshold and membrane potential within the bin is chosen which generates the grid differences:

$$p_{nospikes} = \prod_{t \in t_{grid}} c(\Delta V_{grid}) \tag{38}$$

The log likelihood of the entire spike train being exactly reproduced is given by:

$$LLIN = \log\left(p_{spikes} \times p_{nospikes}\right) = \sum_{t \in t_s} \log(1 - c(\Delta V(t))) + \sum_{t \in t_{grid}} \log(c(\Delta V_{grid})) \quad (39)$$

Simplex Method

The instantaneous threshold, Θ_∞, is optimized to maximize $LLIN$ in Equation 39 using a simplex algorithm (Nelder-Mead). During this procedure Θ_∞ is itself is not optimized, instead, a multiplicative factor (coefficient) is optimized.

To minimize the chance that the optimization routine did not get stuck in a low gradient region, the overall optimization is rerun three times. Each time the overall optimization is rerun, the coefficients are randomly perturbed between an interval of ± 0.3 of their last found optimized value. Within each of the overall reruns, the stability of the convergence is confirmed by reinitializing the algorithm (i.e. re-inflating the simplex) three times at the optimal position in parameter space with a small random perturbation within an interval of $\pm$ 0.01 and then re-running the simplex.

Because the voltage dynamics are governed by a first-order linear differential equation, the voltage dynamics are forward simulated over a single time step using an exact Euler time stepping method. The time step is chosen to be 0.2 ms. The current that forces the dynamics is averaged across the time step.

Changes in instantaneous threshold θ_∞, with optimization

Figure 7 shows how θ_∞ changes with optimization. An estimate of θ_∞ is calculated by measuring the voltage at which a neuron reaches threshold during a 3 ms current pulse. This value is optimized. It is difficult to know exactly why θ_∞ changes in a stereotyped manner, nonetheless, here we speculate.

In general, optimization raises the value of θ_∞ for $GLIF_1$ through $GLIF_4$ and then lowers it in $GLIF_5$ (Supplementary Figure 7 panels c through h). $GLIF_1$ though $GLIF_4$ do not have a voltage dependent threshold, thus, it is likely that θ_∞ is raised to compensate for the lack of voltage dependent threshold that exists in normal neurons. $GLIF_5$ incorporates the voltage dependent threshold; however, there is still not a non-linearity near threshold such as is implemented in the AdEx model [6]. Thus, θ_∞ is probably pulled down to compensate.

Evaluation of Model Spike Times

After all parameters of the model were optimized, the time-evolution of the models was determined using an exact Euler method. The spike times of the model were evaluated against the spike times of the neuron using the 'explained temporal variance' metric described below (Figure 4 of the main text). This metric describes how well the temporal dynamics of the spiking response is captured by the model for a particular level of temporal granularity, Δt.

A spike train is represented as a time series of binary numbers. All numbers in a spike train are zero unless a spike occurs: a spike was denoted with a one. Any spike train can be converted into a single peristimulus time histogram, $stPSTH$, by convolving a spike train with a Gaussian with a standard deviation equal to Δt.

In cases with many repeats of the same stimulus, a peristimulus time histogram (PSTH) could be calculated by taking the mean of the $stPSTH$ at each instant in time. If there were n stimulus repeats and $stPSTH_i$ is denoted with index i with $i = 1, 2...n$, then the PSTH is given by,

$$PSTH = \sum_{i=1}^{n} \frac{stPSTH_i}{n}$$

The variance in spiking output of neurons is described by the variance of the PSTH. The explained variance (EV) between any two PSTHs (multiple or single spike train) is:

$$EV(PSTH_1, PSTH_2) = \frac{var(PSTH_1) + var(PSTH_2) - var(PSTH_1 - PSTH_2)}{var(PSTH_1) + var(PSTH_2)}$$

The explained temporal variance by the mean across-trials $PSTH_D$ of the biological neuron, EV_D is the upper limit on how well the model can perform:

$$EV_D = \frac{1}{n} \sum_{i=1}^{n} EV(stPSTH_i, PSTH_D) \tag{40}$$

where $stPSTH_i$ denotes the ith single trial repeat data PSTH and n denotes the total number of stimulus repeats.

Since the models here were deterministic, the explained variance of the data with the model, EV_{DM}, is calculated by taking the mean of the explained variance between every data $stPSTH_i$ and the model $PSTH_M$.

$$EV_{DM} = \frac{1}{n} \sum_{i=1}^{n} EV(stPSTH_i, PSTH_M) \tag{41}$$

The ratio of the explained variance of the model to the data, EV_{DM}, versus the explained variance within the data, EV_D:

$$EV_{ratio} = \frac{EV_{DM}}{EV_D}. \tag{42}$$

is 0 if the data and the model are independent, and is 1 if the model perfectly predicts the mean of the data.

The EV_{ratio} at a resolution of $\Delta t = 10$ ms (standard deviation of the Gaussian kernel used to convolve the binarized spike times), converted to a percentage is used as the explained variance metric throughout the manuscript (Figure 5 and Table 3 of the main article and Supplementary Figures 6, 8, 9, 10 and Supplementary Table 1). Explained variance is illustrated in Figure 4 of the main article, for the two exemplary neurons reported.

Performance of individual transgenic lines

The explained variance of individual transgenic lines are shown in Supplementary Figures 9 and 10.

Akaike Information Criterion (AIC)

The 'gold standard' method of model selection is to test the model on 'hold out' data as was done throughout the manuscript. Here we supplement the assessment of model performance on 'hold out' data with the calculation of the Akaike Information Criterion (AIC) on the training data. AIC was computed as:

$$AIC = 2k + n \log(RSS/n) \tag{43}$$

where k is the number of variables (1 though 5) at each level, n is the number of independent observations and, RSS is the residual sum of squares. We calculate the RSS between the model spike train convolved with a Gaussian with a standard deviation $\sigma = 10$ ms and the average convolved spike train of the data. n, should be independent samples; here, we use the standard deviation of the Gaussian used for convolution

to calculate an appropriate value for n, $n = T/\sigma$, where T is the total time of a sweep.

In general, the absolute value of the AIC is not important for model selection. Instead, the difference in AIC between two models (Supplementary Figure 11) is often considered:

$$AIC_1 - AIC_2 = 2(k_1 - k_2) + n\log(RSS_1/RSS_2) \tag{44}$$

Clustering

Iterative hierarchical clustering

In order to cluster cells by model parameters or extracted electrophysiological features, we used an iterative binary splitting method using standard hierarchical clustering methods, as follows:

1. Select parameters to cluster on (See Table 2 in the main article).
2. For each parameter, calculate the skew of the distribution and the skew of the distribution of log-transformed values. If the skew of the latter is lower, then use the log-transformed values for that specific parameter.
3. Z-score all parameters (some of which have been log-transformed) and hierarchically cluster cells with Ward's method, using 1 - Pearson's R value as the distance metric.
4. Split cells into two clusters, as defined by the top branch split of the dendrogram from the hierarchical clustering.
5. Train a support vector machine classifier (using a radial kernel) on cell cluster identity using half of the cells, and apply this classifier to generate predicted cell cluster identities for the remaining untrained cells (half of the data), and calculate the total fraction of mis-classified cells in the test set. Repeat this step 100 times, randomly selecting the cells in the training and test sets each time, and identify the maximum mis-classified fraction.
6. If the maximum mis-classified fraction is less than 20 percent, repeat steps 3-5 on each of the two subpopulations, otherwise assign the entire population of cells to a terminal cluster
7. End clustering once all cells are assigned to a terminal cluster that cannot be split further.

Given that the cells are iteratively segregated into a binary tree, the final set of clusters chosen for a given set of parameters can be coherently assembled into a single tree, with each intermediate node being assigned the minimum fraction of correctly predicted cells (using repeated runs of the SVM as described above), as illustrated in the corresponding figure.

We include the confusion matrices between GLIF parameter-based clusters and the electrophysiological feature-based clusters (using the iterative binary splitting algorithm) in Figure 13 of the Supplementary Material based on all 14 parameters.

Affinity propagation

We also applied a second method - affinity propagation - to cluster the cells. Affinity propagation relies on iterative assignment and refinement of clusters using a message-passing algorithm [7]. Briefly, every cell is assigned a responsibility and an availability score with respect to other cells; the former represents how well a given cell serves as an exemplar with respect to every other cell, while the latter represents how well a given cell would be approximated by every other cell as its exemplar. These scores are updated iteratively by passing around both sets of scores until convergence is reached; see [7] for more details. The final number of clusters obtained is nonlinearly dependent on the initial assignment of diagonal values in the

similarity matrix, so we used the gap statistic [8] to determine the final number of clusters for each data set. The clustering results using affinity propagation are shown in Figure 12. The affinity propagation and gap statistic algorithms were run using the apcluster and clusGap packages in R, respectively.

Comparing clusterings

We used two metrics to compare clusterings (all clusterings to Cre line segregation, and all GLIF clusterings to feature-based clustering). The first metric is the Adjusted Rand Index (ARI), which represents the similarity of two partitionings as compared to chance. By definition, an ARI value of 1 indicates perfect agreement and a value of 0 indicates agreement due to chance. The ARI was calculated using the mclust package in R. The second metric is the Adjusted Variation of information (AVOI), which includes a slight modification to the Variation of Information (VOI) metric. The Variation of Information is an extension of mutual information to address the similarity of two parititionings of the same space, and roughly measures the predictability of one clustering based on another. However, the basic VOI measure is sensitive to the total number of clusters, so we report the AVOI, which is the difference between the VOI due to chance (by randomly shuffling the labels of the clusterings 100 times) and the standard VOI of the clusterings being compared. The upper bound for the AVOI is the natural long of the number of cells considered, $ln(645) = 6.47$.

Exclusion Criteria

Here, since we are interested in the models of transgenic lines, we include only cre positive cells and and neurons from transgenic lines that have more than five modelable neurons. Models and neurons were also eliminated if they had inconsistent or insufficient stimuli presented, failed to fit training data, or contained parameters that are biologically unrealistic.

General exclusion criteria are listed below:

1. The training subthreshold noise epochs (used for fitting resistance via linear regression) contained a spike (first epochs in the 'noise 1' stimulus in Figure 2a of the main article).

2. Models with an intercept greater than 30 mV resulting from the linear regression performed during the spike cut width calculation (Supplementary Figure 1).

3. Neurons with an instantaneous threshold, Θ_∞, below -60 mV before post-hoc optimization (Supplementary Figure 7).

4. Models that contain a resistance greater than 1000 $M\Omega$ (Supplementary Figure 2).

5. Neurons which had an inconsistent current amplitude played over noise sweeps.

6. Models which fail to complete the post-hoc optimization without error.

7. The entire model family of a neuron in which one model fails to optimize on training data to an explained variance ratio of at least 20%.

Additional neurons were eliminated from models containing reset rules based on the following criteria:

1. Models which had an amplitude of the spike component of threshold, δ_s, that was larger than 20 mV (Supplementary Figure 3).

2. Models which had an amplitude of the spike component of threshold, δ_s that was less than zero (Supplementary Figure 3)

3. Models which had a $1/b_s$ that was greater than 100 mS^{-1} (Supplementary Figure 3).

Finally models with the following voltage dependent threshold are excluded:

1. Models which had a_v less than -50 s^{-1} (Supplementary Figure 3).
2. Models which had a b_v is less than 0.1 s^{-1} (Supplementary Figure 3).

This leaves a total of 645 neurons that have a $GLIF_1$, and $GLIF_3$ model, 254 neurons that have a $GLIF_2$ and a $GLIF_4$ model, and 253 neurons that have a $GLIF_5$ model (Table 2 of the main article). Note the large decrease in models for $GLIF_2$, $GLIF_4$ and $GLIF_5$ is not due to exclusions. Instead, for reasons unrelated to this study, the triple short square stimulus (Stimulus section in the Methods of the main article, Figure 2 of the main article and Supplementary Figure 3) necessary for these model levels was not played to approximately half of the neurons.

References

[1] Allen Institute for Brain Science. Allen Cell Types Database, Technical White Paper: Electrophysiology (2016).

[2] Pozzorini, C. *et al.* Automated high-throughput characterization of single neurons by means of simplified spiking models. *PLoS Comput Biol* **11**, e1004275 (2015).

[3] Paninski, L., Pillow, J. W. & Simoncelli, E. P. Maximum likelihood estimation of a stochastic integrate-and-fire neural encoding model. *Neural computation* **16**, 2533–2561 (2004).

[4] Dong, Y., Mihalas, S., Russell, A., Etienne-Cummings, R. & Niebur, E. Estimating parameters of generalized integrate-and-fire neurons from the maximum likelihood of spike trains. *Neural computation* **23**, 2833–2867 (2011).

[5] Mensi, S. *et al.* Parameter extraction and classification of three cortical neuron types reveals two distinct adaptation mechanisms. *Journal of neurophysiology* **107**, 1756–1775 (2012).

[6] Brette, R. & Gerstner, W. Adaptive exponential integrate-and-fire model as an effective description of neuronal activity. *Journal of neurophysiology* **94**, 3637–3642 (2005).

[7] Frey, B. J. & Dueck, D. Clustering by passing messages between data points. *Science* **315**, 972–976 (2007).

[8] Tibshirani, R., Walther, G. & Hastie, T. Estimating the number of clusters in a data set via the gap statistic. *Journal of the Royal Statistical Society: Series B* **63**, 411–423 (2001).
